# Probing Jahn–Teller Distortions and Antisite Defects in LiNiO_2_ with ^7^Li NMR Spectroscopy and Density Functional Theory

**DOI:** 10.1021/acs.chemmater.3c03103

**Published:** 2024-04-24

**Authors:** Annalena R. Genreith-Schriever, Chloe S. Coates, Katharina Märker, Ieuan D. Seymour, Euan N. Bassey, Clare P. Grey

**Affiliations:** † Yusuf Hamied Department of Chemistry, University of Cambridge, Cambridge CB2 1EW, U.K.; ‡ Univ. Grenoble Alpes, CEA, IRIG, MEM, Grenoble 38000, France; § Department of Materials, Imperial College London, London SW7 2AZ, U.K.; ∥ Department of Chemistry, School of Natural and Computing Sciences, University of Aberdeen, Aberdeen AB24 3FX, U.K.; ⊥ Advanced Centre for Energy and Sustainability, School of Natural and Computing Sciences, University of Aberdeen, Aberdeen AB24 3FX, U.K.; # The Faraday Institution, Harwell Science and Innovation Campus, Didcot OX11 0RA, U.K.

## Abstract

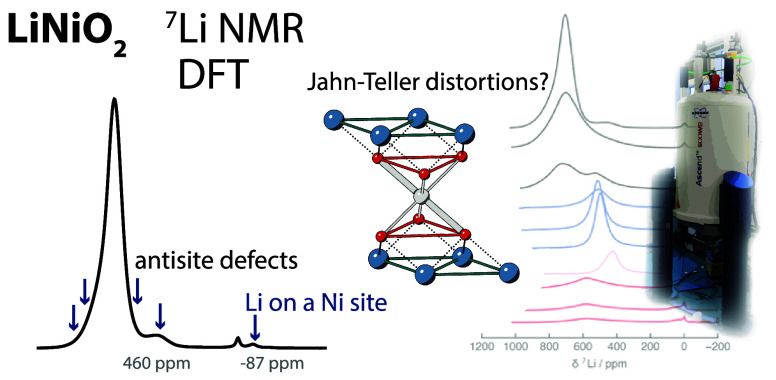

The long- and local-range structure and electronic properties of the high-voltage lithium-ion cathode material for Li-ion batteries, LiNiO_2_, remain widely debated, as are the degradation phenomena at high states of delithiation, limiting the more widespread use of this material. In particular, the local structural environment and the role of Jahn–Teller distortions are unclear, as are the interplay of distortions and point defects and their influence on cycling behavior. Here, we use *ex situ*^7^Li NMR measurements in combination with density functional theory (DFT) calculations to examine Jahn–Teller distortions and antisite defects in LiNiO_2_. We calculate the ^7^Li Fermi contact shifts for the Jahn–Teller distorted and undistorted structures, the experimental ^7^Li room-temperature spectrum being ascribed to an appropriately weighted time average of the rapidly fluctuating structure comprising collinear, zigzag, and undistorted domains. The ^7^Li NMR spectra are sensitive to the nature and distribution of antisite defects, and in combination with DFT calculations of different configurations, we show that the ^7^Li resonance at approximately −87 ppm is characteristic of a subset of Li–Ni antisite defects, and more specifically, a Li^+^ ion in the Ni layer that does not have an associated Ni ion in the Li layer in its 2nd cation coordination shell. *Via ex situ*^7^Li MAS NMR, X-ray diffraction, and electrochemical experiments, we identify the ^7^Li spectral signatures of the different crystallographic phases on delithiation. The results imply fast Li-ion dynamics in the monoclinic phase and indicate that the hexagonal H3 phase near the end of charge is largely devoid of Li.

## Introduction

Lithium nickel oxide (LNO) is a layered oxide material which remains of fundamental interest both for its unusual physics as well as for practical application in lithium-ion batteries.^1−3^ It is the parent compound of the commercially relevant NMC (LiNi_*x*_Co_*y*_Mn_1–*x*–*y*_O_2_) and NCA (LiNi_*x*_Co_*y*_Al_1–*x*–*y*_O_2_) families of positive electrode materials. While LNO has a high practical capacity of around 250 mAh g^–1^ and has fewer mining and cost concerns, as compared to the prototypical and isostructural LiCoO_2_ cathode material, it suffers from more severe capacity degradation, attributed, in part, to the instability of Ni^4+^ (leading to oxygen loss^4^), coupled with particle cracking from phase-transformation-induced stresses on electrochemical cycling.^5^

**Figure 1:**
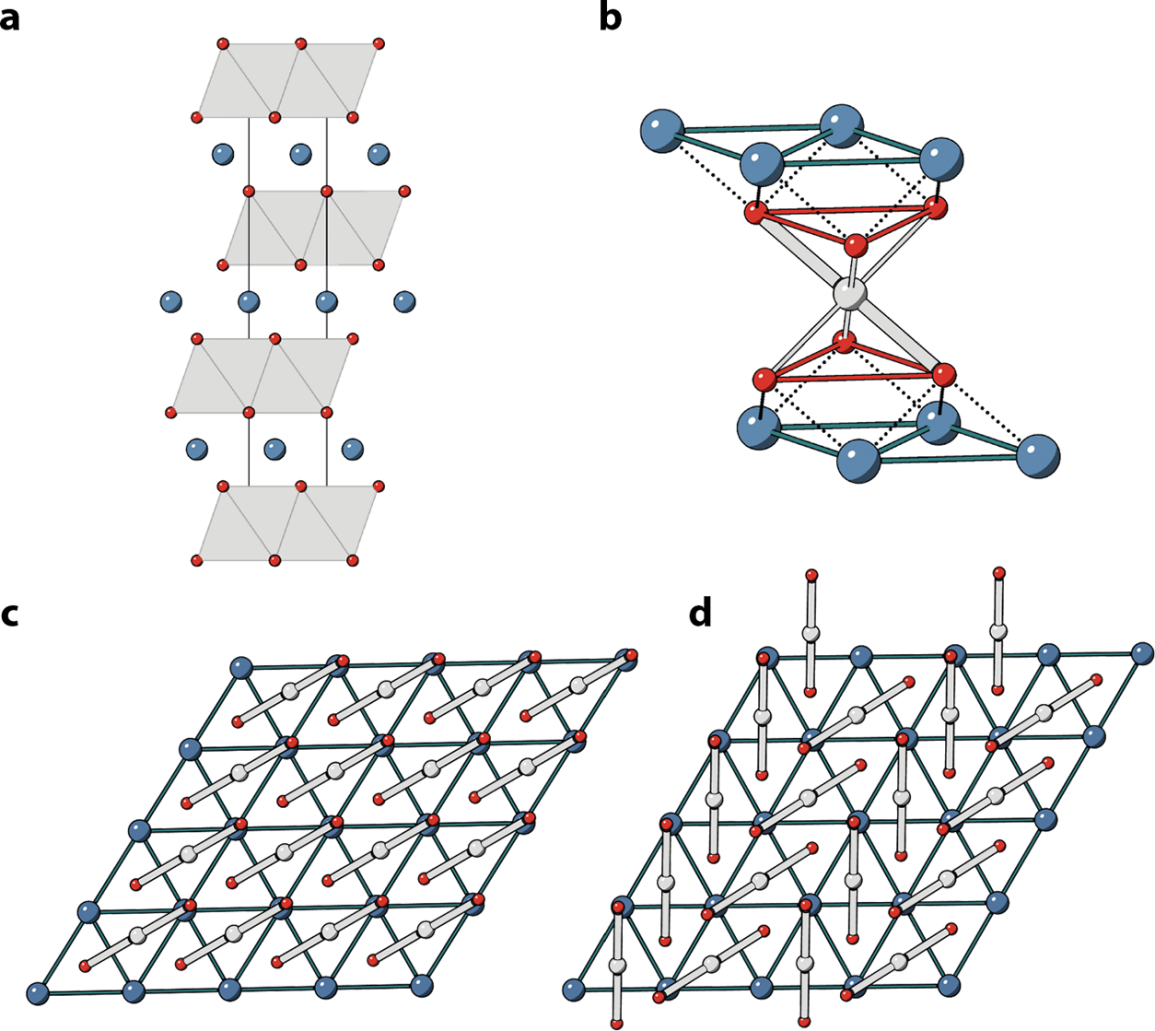
Structure of LiNiO_2_. (a) ABC stacking of Li, Ni, and O layers with the unit cell of *R*3̅*m* structure shown along along the hexagonal **a** axis; Li in blue, Ni in gray, and O in red. (b) Experiments and theory suggest that the Ni^3+^ ions are locally Jahn–Teller distorted at low temperatures^12,13^ (<250 K for stoichiometric LNO). Each distortion breaks the 3-fold symmetry *via* elongation along one of the local O–Ni–O axes, resulting in four short (thin gray lines) bonds and two long bonds (thick gray lines). View down the hexagonal **c** axis for the (c) collinear (*C*2/*m*) and (d) zigzag (*P*2_1_/*c*) arrangements of Jahn–Teller distortions, which are both predicted by DFT to be more stable than the experimentally observed *R*3̅*m* structure.

The average (bulk) structure of lithium nickel oxide has rhombohedral symmetry denoted by the crystallographic space group *R*3̅*m*, typical of layered Li-ion cathode materials, including LiCoO_2_, as shown in Figure 1a. For LNO, this structure is also referred to as the H1 phase (the first hexagonal phase, also known as the O3 phase, to illustrate octahedral coordination and ABC stacking of the O sublattice).^1,6,7^ There is experimental evidence from extended X-ray absorption fine structure (EXAFS) and low-temperature neutron pair distribution function (PDF) measurements of local Jahn–Teller distortions of the NiO_6_ octahedra—resulting in four short and two long Ni–O bonds—as a result of the formal d^7^ (t_2g_^6^e_g_^1^) electronic configuration of Ni^3+^ (Figure 1b).^12,13^ The average *R*3̅*m* structure is, however, incompatible with a static cooperative Jahn–Teller distortion, as is observed in the related system NaNiO_2_, in which the long Jahn–Teller axes align in a collinear arrangement to give *C*2/*m* symmetry (Figure 1c).^10,11^

Density functional theory (DFT) studies have consistently predicted ground states for pristine LNO that involve cooperative Jahn–Teller distortions, and thus long-range symmetry that is lower than *R*3̅*m*.^8,9,14,16^ Row-orderings of Jahn–Teller distortions are generally predicted to be more stable.^14^ Furthermore, both collinear and zigzag orderings (Figure 1c,d) are calculated to be much lower in energy than either undistorted rhombohedral, other (e.g., trimer) orderings or charge disproportionated LNO, where Ni is predicted to occur in charge states of Ni^2+^ and Ni^4+^.^8,9,14−16^ Note that there is an ongoing discussion regarding the possibility of charge-transfer effects in LNO, leading to electron holes on O and the Ni charges to deviate from 3+.^4,15^ This discussion clarifies how electron density is distributed between Ni and O but does not affect the overall Ni–O spin (i.e., how much unpaired spin density is present among Ni and O); for the sake of simplicity, to distinguish between the spin states, we will adhere to convention here and label the states according to their formal oxidation states.

The seeming incompatibility of the local distorted and average undistorted symmetry has been attributed variously to small randomly oriented domains of static ordered distortions, ordered layers with weak interlayer correlations, and dynamic Jahn–Teller distortions.^12,14,16−18^ The general consensus, however, is that the Jahn–Teller distortions in LiNiO_2_ are dynamic at room temperature, with a change of the long Jahn–Teller axis occurring with a frequency of greater than 10^11^–10^12^ Hz above 100 K, as shown from both electron-spin resonance (ESR) measurements and molecular dynamics (MD) simulations.^16,18−20^ The dynamic behavior has traditionally been explained in terms of pseudorotations of the Ni–O long axes in a disordered high-temperature phase.^16^ We have recently proposed that the dynamic behavior is instead caused by the onset of a biphasic phase transition to a high-temperature so-called “displacive phase,” a term coined by Radin et al.^21^ (biphasic regime at 250 < *T* < 350 K).^18^ The recurring formation of Jahn–Teller distorted and undistorted domains in the biphasic regime allows the Jahn–Teller long axes to reorient at room temperature with frequencies that approach a THz. Above 350 K, the material assumes a highly dynamic, displacive phase in which the lattice vibrations are so strong that the NiO_6_ octahedra spend most time in or near undistorted configurations.^18^^6/7^Li nuclear magnetic resonance spectroscopy (NMR) is a powerful tool to probe the local structural environment in layered oxides containing paramagnetic ions, shedding light on Li dynamics, ordering phenomena, and structural distortions in battery cathode materials.^22−26^ The time scale of Jahn–Teller pseudorotations reported for LNO in the biphasic regime (250 < *T* < 350 K) is faster than the time scale probed by NMR (1–10^9^ Hz), and thus a time-averaged and appropriately weighted signal is likely to be observed in the NMR spectrum for the defect-free material, as explored further below.^18,27^

The experimental hyperfine or Fermi contact (FC) shift values, *i.e.*, those caused by spin density transfer from the paramagnetic ion (Ni^3+^) *via* O to the Li *s*-orbitals, obtained for LiNiO_2_ can be rationalized in the context of Jahn–Teller distortions, using density functional theory (DFT) calculations.^27^ Using an optimized cell of cooperatively distorted, collinear LNO (space group *C*2/*m* as in Figure 1c), Middlemiss et al. used a spin-flip approach to determine the individual bond pathways and their contribution to the FC shift.^27^ Each Li has six nearest-neighbor Ni^3+^ ions—connected *via* oxygen with 90° Li–O–Ni^3+^ superexchange interactions—and six next-nearest-neighbor Ni^3+^, which experience 180° Li–O–Ni^3+^ interactions. The additional six nearest-neighbor Li ions (90° Li–O–Li) within the same layer have no unpaired electrons, and so do not contribute to the FC shift. The nature of the superexchange interaction depends on the relative orientation of the Jahn–Teller distortion, *i.e.*, whether the interaction occurs *via* a long or short Ni–O bond, with the 180° interaction *via* a long JT-distorted Ni–O bond having the most significant effect on the shift (a contribution of +326 ppm *vs.* a smaller contribution from the 180° interaction *via* a short JT-distorted Ni–O bond of +44 ppm, based on hybrid DFT calculations with 20% Fock exchange).^27^ The shifts of dynamic Jahn–Teller distortions were then predicted by averaging the static shifts obtained from the bond-pathway analysis.

One key aspect that complicates a range of observations in LNO is the seemingly ubiquitous presence of antisite mixing, that is, the presence of Ni in the Li layer (Ni_Li_) and Li in the Ni layer (Li_Ni_). Nonstoichiometry with excess Ni is also possible.^28^ Ni_Li_ is generally thought to exist as formally Ni^2+^ due to its larger radius (in comparison to Ni^3+^), which is closer to that of Li^+^. This mixing impacts the physical properties, including magnetism, phase transformations on cycling, decomposition temperature, and capacity retention.^3,29−31^ Not only is it extremely difficult to control the degree of antisite defects during synthesis, but it is also challenging to characterize accurately. For example, the ratio of Li/Ni on each crystallographic site is hard to access using Rietveld refinements, since the occupancies are highly correlated with atomic displacement parameters and oxygen positions. Recent work by Nguyen et al. has also considered defects beyond the antisite defect and has explored the different Li and Ni local environments in planar defects (twin boundaries).^32^

It is possible to estimate the degree of antisite mixing *via* the magnetic transition temperature and Weiss constant.^28^ LiNiO_2_ undergoes an antiferromagnetic ordering transition with a Néel temperature of approximately *T*_N_ = 9 K for the most stoichiometric samples with *x* = 0.004 in Li_1–*x*_Ni_*x*_NiO_2_, the temperature of which increases with increasing Ni_Li_.^28,32^ In NaNiO_2_, which can be prepared without antisite mixing, there is an antiferromagnetic ordering transition at *T*_N_ = 20 K;^33^ the magnetic moments are ferromagnetically aligned within the layers and antiferromagnetically aligned between the layers. The same intralayer ferromagnetic interactions are understood to exist in LiNiO_2_; however, the presence of Ni_Li_ defects results in strong antiferromagnetic superexchange interactions between Ni^3+^–O–Ni_Li_^2+^ (as determined by the Goodenough–Kanamori rules) inducing strong ferromagnetic interactions between neighboring layers and resulting in ferrimagnetic clusters and frustrating longer-range antiferromagnetic interactions.^29,34^ NMR spectroscopy is also sensitive to defects; Nguyen et al., for example, have used DFT calculations to help assign the observed weaker ^7^Li LNO resonances to Li sites near the grain boundary and in the Ni layers.^32^

LNO undergoes a series of reversible structural transformations on delithiation: H1–M–H2–H3, where H1, H2, and H3 are hexagonal layered structures with space group *R*3̅*m* with distinct O *c*-parameter ranges^1,7,35^ and M is monoclinic (with space group *C*2/*m*).^7,36^ The H2 to H3 transition, in particular, is associated with a large lattice collapse along *c*, and the resulting lattice strain is thought to be responsible for the cracking-induced degradation.^3^ These transformations have been widely studied using X-ray diffraction (XRD), X-ray absorption (XAS), NMR, and electron diffraction, using both *ex situ* and *operando* characterization.^5,37−42^

Previous ^6/7^Li measurements of LNO at different states of lithiation have explained the observed FC shifts by considering the predicted vacancy-ordered ground states.^23,32,37,43^ This requires a detailed understanding of the interplay of Ni^4+^, the relative orientation of JT axes, and potential Li-ion and/or JT dynamics. This has been challenging, and in many cases, the agreement between experiment and calculations is poor: neither the estimated shifts based on the Li–O–Ni^3+^ bond pathways with given vacancy orderings nor the shifts from the average oxidation state give a good estimate of the shift. The exception is for samples at high states of delithiation; for Li_0.25_NiO_2_, the high measured FC shift of approximately 600 ppm is consistent with vacancy ordering and chains of Ni^3+^–O–Li^+^ (averaging of the shifts calculated for bond pathways involving 25% Ni^3+^ and 75% Ni^4+^ would give a predicted shift of only approximately 142 ppm).^23,27^

Here, we extend previous ^7^Li NMR measurements and DFT calculations for lithium nickel oxide to include calculations for the ground-state zigzag structure and to consider the role that antisite defects have on the NMR spectra. We demonstrate that NMR is extremely sensitive to the nature of the antisite defects in LNO. Our sample contains both Ni in the Li layer and Li in the Ni layer, and we show that the latter is either removed on charging or forms next-nearest-neighbor configurations with the Ni in the Li layer in Ni-rich environments. We present and interpret the high-resolution *ex situ*^7^Li NMR data as a function of the state of charge, identifying the spectral signatures of the different crystallographic phases on delithiation. These reveal fast Li-ion dynamics in the monoclinic phase and Li deficiency of the H3 phase.

## Materials and Methods

### Materials

LNO powder was obtained from BASF. For *ex situ* characterization of cycled samples, LNO electrodes were prepared from 90 wt % LNO (BASF), 5 wt % conductive carbon (Timcal C45), and 5 wt % poly(vinylidene difluoride) (PVDF) binder (Solvay 5130). The LNO electrodes were assembled into LNO/Li half-cells in 2032 coin cells (Cambridge Energy Solutions), consisting of one ^1^/_2_ in. cathode with a thickness of 150 μm, one 5/8 in. glass fiber separator (GF/B, Whatman) soaked with 150 μL LP30 electrolyte, and one Li metal disk. A steel spring and two steel spacers were used to maintain pressure. The electrolyte used was LP30 (1.0 M LiPF_6_, ethylene carbonate (EC)/dimethyl carbonate (DMC) 1:1 v/v, battery grade, Sigma-Aldrich). Li metal (99.95%) disks were purchased from LTS Research Laboratories, Inc. All procedures described were performed in an argon-filled glovebox with water and oxygen levels below 10 ppm.

### NMR and XRD Sample Preparation of Partially Charged LNO

The sample preparation for *ex situ* characterization involved galvanostatically cycling LNO/Li half-cells at a rate of 10 mA g^–1^ (C/20 for an estimated practical capacity of 200 mAh g^–1^). The cycling was stopped at a predefined potential (all electrochemical potentials given *vs*. Li/Li^+^) and held for 12 h to allow for equilibration. After cycling, the coin cells were disassembled within 15 min under an argon atmosphere to extract the cathode. The cathode was washed with DMC (*ca.* 1 mL) and dried under vacuum for 30 min. The cathode material was then scraped off the current collector and packed into a 1.3 mm magic-angle spinning (MAS) ZrO_2_ NMR rotor, with the NMR samples weighing between 3.0 and 4.3 mg. 8.0 mg of pristine LNO powder (BASF) was also packed into a 1.3 mm MAS NMR rotor.

### *Ex Situ* Solid-State NMR Experiments

Solid-state NMR experiments were performed on a 4.7 T (200 MHz ^1^H Larmor frequency) Bruker Avance III spectrometer using a Bruker 1.3 mm double-resonance probe. Spectra were acquired at a MAS frequency of 60 kHz, and ^7^Li radiofrequency (rf) pulses were applied at ∼230 kHz rf field strength. Recycle delays between 30 and 77 ms were employed, which were quantitative for the bulk signal of LNO (but not for the signals of diamagnetic Li salts at ∼0 ppm). Spectra were measured using room-temperature spinning gases, but at the MAS frequencies used here, the actual sample temperature is typically between 50 and 55 °C. Experiments on pristine LNO were also performed with temperature sensor readings ranging 25–60 °C (see the Supporting Information (SI)). ^7^Li chemical shifts were referenced externally using Li_2_CO_3_ (0 ppm). Projection MATPASS spectra^44^ were recorded with eight *t*_1_ increments and between 25,600 and 102,400 scans per *t*_1_ increment, depending on the Li content of the sample. All spectra presented in the main paper correspond to the central slice of the respective projection MATPASS spectrum and are scaled by sample mass and number of scans unless stated otherwise. A standard automated baseline correction was used as implemented in Bruker’s Topspin software using a fifth-degree polynomial.

Spin–lattice, *T*_1_, relaxation times were measured using saturation–recovery experiments and fitted using a single *T*_1_ component. Spin–spin, *T*_2_, times were measured with a rotor-synchronized Hahn echo sequence, by varying the evolution times.

#### X-ray Diffraction Experiments

Laboratory X-ray diffraction experiments were carried out using a PANalytical Empyrean diffractometer (Cu Kα radiation, λ = 1.541 Å) of both the pristine and cycled samples. Cycled cathode electrodes, prepared for *ex situ* NMR experiments, were mounted between Kapton sheets under an argon atmosphere before being transferred to the diffractometer, to ensure minimal exposure to air. Diffraction patterns were recorded between 2θ = 5–80°. All Rietveld refinements were carried out using TOPAS Academic v. 6.

#### Density Functional Theory Calculations

Density functional calculations were performed with the all-electron CRYSTAL software package^45^ using the hybrid functional B3LYP with 20% Fock exchange. The basis sets proposed by Bredow and coworkers were used^46^ on supercells comprising 64–128 ions (2 × 2 × 2 and 2 × 4 × 2 supercells of the zigzag distored structure, and 2 × 2 × 2 supercells of the undistorted structure). Geometry optimizations were performed until the energies differed by no more than 10^–6^ eV and forces no more than 0.001 eV/Å. A Monkhorst–Pack *k*-point grid of 2 × 2 × 2 was chosen for the geometry optimizations. Single-point calculations of the energies and the spin density at the nucleus, decisive for the Fermi contact shift, were performed with a finer *k*-point grid. The nuclear spin density showed convergence with respect to the *k*-points at 2 × 4 × 2 *k*-points for the distorted smaller cells, *i.e.*, the cell size requiring the largest number of *k*-points (at a grid density of 20 × 24 × 20 Å^3^) (see the SI). The hyperfine coupling constant and Fermi contact shift were calculated from the nuclear spin density according to Kim et al.^47^ and scaled to 320 K (while the experiment was nominally conducted at room temperature, frictional heating of the rotor results in a sample temperature of *ca.* 320 K) using Curie–Weiss parameters, as reported by Mukai and Sugiyama.^48^ For the selected computing parameters, a range of hybrid functionals was tested, including HSE06 and PBE0, yielding excellent agreement of the predicted Fermi contact shifts (with shift differences of <20 ppm, see the SI).

## Results

### Uncycled LNO

#### XRD and NMR

The average structure of LiNiO_2_ was refined, using laboratory-based XRD data, as the H1 phase using *R*3̅*m* symmetry^1,7^ (see the SI for Rietveld refinements), accounting for the Li/Ni occupancies to quantify (approximately) the level of antisite mixing. To minimize covariance between Li/Ni occupancies and atomic displacement parameters, isotropic atomic displacement parameters were fixed to sensible values,^3^ and Li/Ni occupancies were allowed to refine on both the Li and Ni sites. This resulted in an estimate of 3.8(6)% Li on the Ni site and 3(1)% Ni on the Li site. Within error, this LiNiO_2_ sample corresponds to a sample with antisite mixing only (of approximately 3%) rather than a Ni-excess sample (*i.e.*, one with a Ni/Li ratio of more than 1).

**Figure 2:**
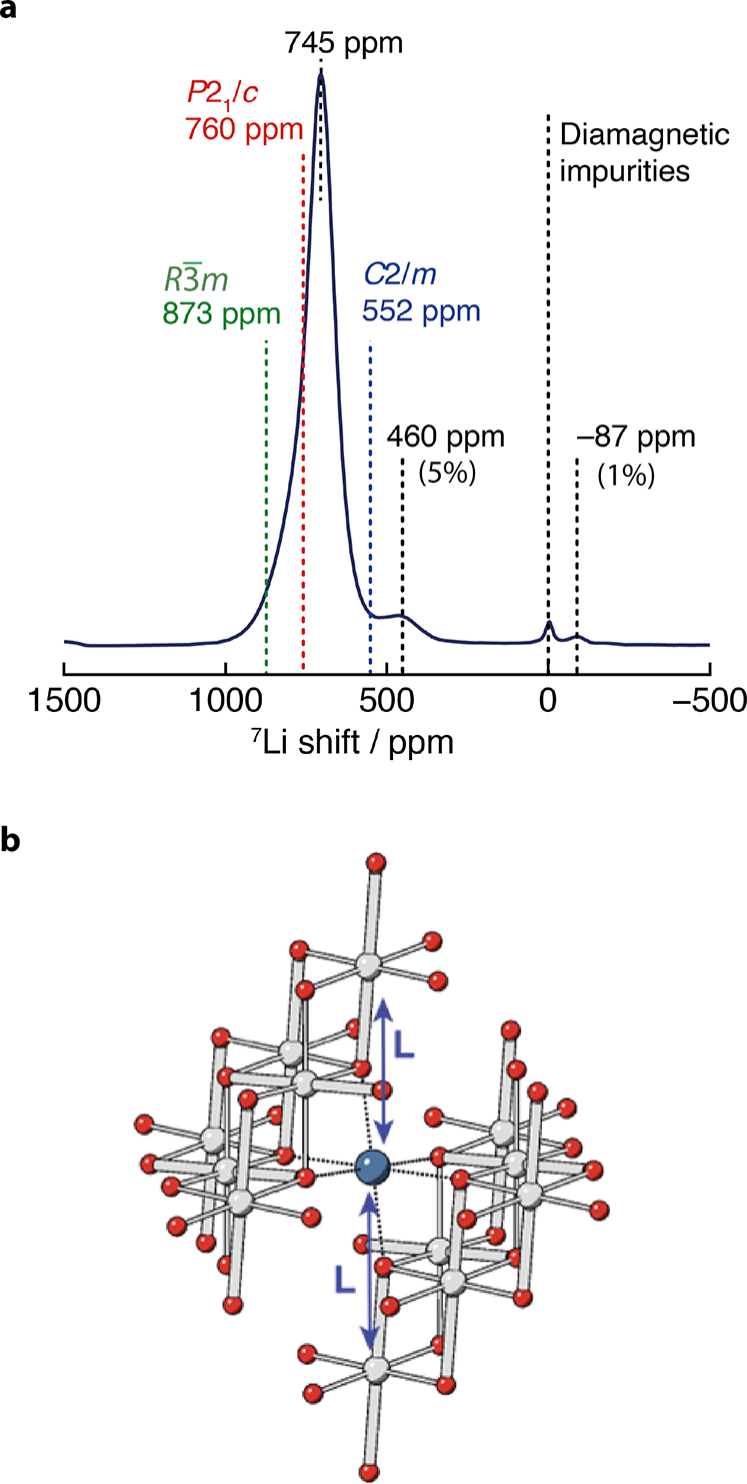
(a) ^7^Li MAS NMR spectrum of uncycled LNO at room temperature. The major isotropic resonances are marked with their shifts, with the intensities (%) relative to the main peak given for the two peaks arising from defects. The dashed lines represent diamagnetic impurities (black) and calculated shifts for collinear (*C*2/*m*, blue), zigzag (*P*2_1_/*c*, red), and undistorted (*R*3̅*m*, green) LNO. (b) Local Li environment for zigzag LNO. Li in blue, Ni in gray, and O in red. Each distortion breaks the 3-fold symmetry *via* elongation along one of the local O–Ni–O axes (thick gray lines), resulting in 2 long Ni–O–Li bonds (L) and 4 short Ni–O–Li (thin) bonds contributing to the overall shift.

Four paramagnetically shifted peaks (with isotropic resonances marked on the spectrum) can be identified in the ^7^Li NMR spectrum of our pristine LNO sample (Figure 2a), consistent with findings by Nguyen et al.,^32^ Karger et al.,^49^ Carlier et al.,^50^ and Li et al.;^37^ peak fitting and proportions are included in the SI (Figure S4a). The peak at 0 ppm is present in all samples and represents a small amount of diamagnetic Li from surface diamagnetic impurity phases, *e.g.*, Li_2_CO_3_, LiHCO_3_, and LiOH. The most intense resonance at 745 ppm is consistent with Li in a paramagnetic environment with 180 and 90° superexchange interactions with Jahn–Teller distorted Ni^3+^ occurring *via* intervening O.^27,32,37^ This resonance is asymmetric and best fit using (at least) two peaks (see Figure S4b,c); the origin of the asymmetry of this peak remains an open question that we address below (in the Section “Antisite Mixing”). It has been found empirically that Ni^2+^ overstoichiometry can also increase the broadening and asymmetry of this peak.^22^ We now explore what insights DFT calculations can offer into the line shape and hyperfine shifts associated with the main 745 ppm resonance.

#### DFT

**Figure 3:**
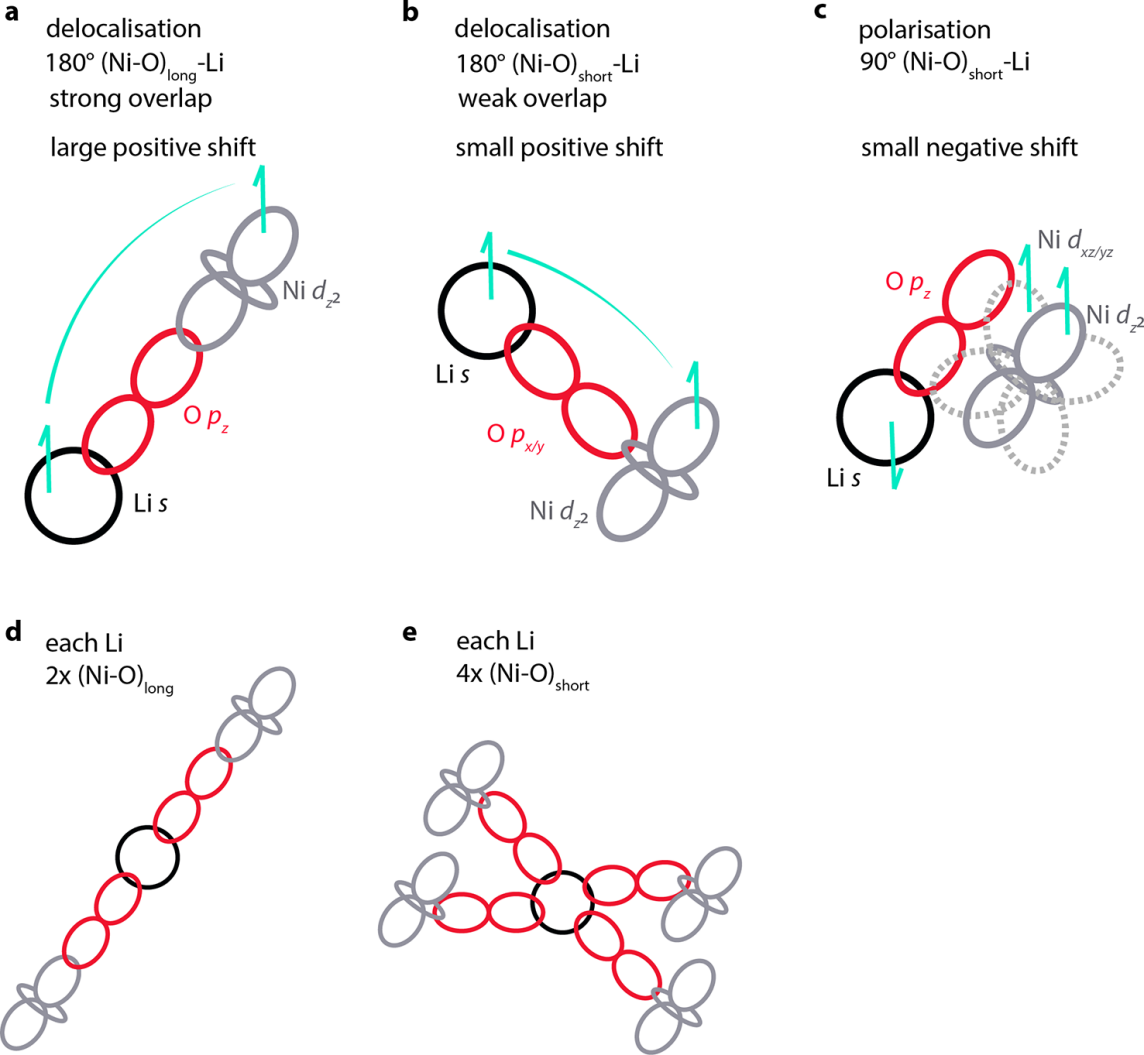
Dominant spin transfer mechanisms accounting for the Fermi contact shifts in LNO. (a) 180° Ni–O–Li interaction of Ni d_*z*^2^_–O p_*z*_–Li s *via* a long Ni–O bond: delocalization with strong overlap resulting in a strongly positive shift; (b) 180° Ni–O–Li interaction of Ni d_*z*^2^_–O p_*x*/*y*_–Li s *via* a short Ni–O bond: delocalization with weak overlap resulting in a weakly positive shift; and (c) 90° Ni–O–Li interaction of Ni d_*z*^2^_ polarizing doubly occupied Ni t_2g_ states, O p_*z*_–Li s: inducing negative spin density at Li and resulting in a weakly negative shift. Each Li ion interacts with the nearest Ni ions through (d) two long Ni–O bonds and (e) four short Ni–O bonds.

The previous Fermi contact shift calculations of a collinear cooperatively distorted cell with *C*2/*m* symmetry^27^ were first reproduced before calculating the expected shifts for the more stable (at 0 K) zigzag arrangement of Jahn–Teller distortions (although both configurations are expected to be accessible at room temperature as well as undistorted domains).^18,27^ The zigzag structure was then used as a starting point to explore the effect of Li_Ni_ and Ni_Li_ antisite defects on the NMR spectrum. The expected ^7^Li shifts for the zigzag (*P*2_1_/*c*), collinear (*C*2/*m*), and undistorted LNO (*R*3̅*m*) structures are shown in Figure 2, alongside the local Li environment for the zigzag cell. Each Li experiences two 180° Li–O–Ni interactions (Figures 2b and 3a) with Li s–O p_*z*_–Ni d_*z*^2^_*via* a long Ni–O bond, the spin delocalization mechanism with a strong orbital overlap resulting in a strongly positive shift. Four 180° Li–O–Ni interactions through short Ni–O bonds give rise to small positive contributions to the spin: the unoccupied Ni d_*x*^2^–*y*^2^_ orbital now points toward the O in the short Ni–O, and the overlap of the occupied Ni d_*z*^2^_ orbital and the O p_*x*_ and O p_*y*_ orbitals is much smaller than that of the Ni d_*z*^2^_ and the O p_*z*_ orbitals in the case of a long Ni–O bond (Figure 3b). Negative contributions to the shift are expected for the 90° Li–O–Ni interaction (see Figure 3c) *via* the polarization of the doubly occupied Ni d_*xz*_/d_*yz*_ orbitals by the Ni d_*z*^2^_ electron, which in turn leads to an accumulation of positive spin density of the O p_*z*_ states closer to the Ni center, and negative spin density at the Li position.

Both the zigzag and the collinear simulation cells were geometry-optimized, and FC shifts of 760 and 552 ppm were calculated for the zigzag and collinear cells, respectively. The undistorted *R*3̅*m* structure, however—being an unfavorable structure at 0 K—relaxes into a distorted structure if geometry-optimized. For this reason, a single-point calculation was performed, fixing the lattice parameters to experimentally reported values.^7^ While the shifts of the distorted structures quickly converged with respect to the choice of *k*-point grid, the *R*3̅*m* cell required a much higher *k*-point density. For a 2 × 2 × 2 *k*-point grid (with a grid density of *ca.* 12 × 12 × 57 Å^3^), shifts of *ca.* 2000 ppm were determined, in line with shifts previously reported for the undistorted phase.^27^ The shifts, however, decreased drastically to *ca.* 873 ppm when increasing the *k*-point density to 6 × 6 × 3 *k*-points (with a grid density of *ca.* 35 × 35 × 85 Å^3^), the value approaching those of the distorted phases. The larger shifts calculated for the *R*3̅*m* cell reflect the two shorter Ni–O bonds compared to the long Ni–O bonds in the distorted phase; this leads to larger O p_*z*_–Ni d_*z*^2^_ overlap in the *R*3̅*m* cell and thus larger spin transfer. The larger magnitude for the Li shift of the zigzag *vs.* the collinear cell is similarly ascribed to the smaller volume of the geometry-optimized zigzag cell. We note that a single Li environment is present for all three space groups and that this environment is similar for both the collinear and zigzag cells, with two long and four short Ni–O bonds pointing toward the Li. Radin et al. demonstrated that all permutations of row-orderings (zigzag, zigzigzag, *etc.*) are close in energy, within 40 meV per formula unit,^14^ so we might expect dynamics and fluctuations between these different arrangements both within and between layers if the octahedra change direction. We have furthermore observed *via* AIMD simulations the formation of domains without Jahn–Teller distortions at temperatures between 250 and 350 K,^18^*i.e.*, we expect fluctuations not only between different row-orderings of Jahn–Teller distortions but also between distorted and undistorted domains. The question then arises if we can predict the NMR shifts of Li in the dynamically distorted/undistorted material at room temperature based on static DFT calculations. To address this, we will first consider a (likely hypothetical^18^) scenario of dynamic distortions without undistorted domains to assess whether static DFT calculations can capture the NMR shifts of the dynamic distortions. Building on this, we will then explore whether the DFT calculations can predict the shifts of experimental samples where the dynamic distortions not only fluctuate between different ordering types but also between Jahn–Teller distorted and undistorted states.^18^ When a system undergoes structural changes on a time scale shorter than the NMR time scale, the experimentally observed shift is typically a weighted average of the different configurations. Computational approaches have been proposed to average the predicted shifts accordingly.^27^ In the case of LNO, if all directions of a long Ni–O bond are equally likely, the experimental spectrum of the dynamically distorted material simplifies to resemble approximately the spectrum of the statically distorted material. Let us illustrate this by analyzing the model scenario where a system is dynamically distorted and the distortions are fully correlated. After a pseudorotation of all octahedra, the absolute orientation of the Ni–O long bonds relative to an external frame of reference is changed, but the local Li environment that NMR spectroscopy probes is unaffected, giving the same isotropic shift and intensity for the main peaks. The only additional (isotropic shift) information that could be gained from a dynamic computational treatment of the shifts are contributions to the shifts arising from the system spending brief times in transition states when the long axis changes orientation. Depending on the nature of the transition state and the time the structure spends in the transition state, these may emerge as clearly distinguishable additional peaks, cause broadening, or, in this case (where JT fluctuations approach the THz regime), cause very small shifts to the time-averaged isotropic resonance. The main peaks, however, are expected to be essentially identical in the cooperative static and cooperative dynamic case if the extent of the distortion is similar. We note that the fluctuations may affect measurements that are sensitive to dynamics, including the ^7^Li spin–lattice (*T*_1_) and spin–spin (*T*_2_) relaxation times, depending on their time scales; anisotropic interactions such as the ^7^Li quadrupolar interaction and dipolar interactions between nuclei and between unpaired electrons and nuclei may also be modified.

Next, we remove the constraint of cooperativity and consider the case where each octahedron can rotate freely, pointing randomly into one of the three possible directions. A range of 1–6 long Ni–O bonds could now point toward each Li. If the local, uncorrelated orientations were frozen in, a distribution of Li shifts would be expected, whose magnitude would be governed by the number of long bonds pointing toward the Li ion.^27^ In the case of local, dynamic distortions, however, only the time average of the number of long bonds is of relevance. If all directions are equally likely, the average number of long Ni–O bonds pointing toward the Li ion in the local dynamic case is 2, as in the static cooperative case. The shifts determined for the static cooperative case thus directly correspond to the main peaks in the dynamically distorted material, irrespective of the degree of cooperativity, *i.e.*, irrespective of the domain size of ordered distortions (note that shifts in frequency can be expected if the domain size affects the unit cell volume, *i.e.*, if the size of the domains modifies the orbital overlap and thus amount of spin density transferred to the Li ions). Static DFT calculations can therefore serve to predict the NMR shifts of dynamically distorted LiNiO_2_. Having established this, we can turn to the second part of our question and explore whether static DFT calculations can serve to predict the shifts of experimental samples of LiNiO_2_ at room temperature, where we expect coexistent Jahn–Teller undistorted and distorted domains (presumably fluctuating between different orderings).^18^ The local Li environment is very similar in all cases, but the slightly different lattice parameters of the collinear, zigzag, and undistorted structures result in different degrees of orbital overlap, causing slight variations in the Fermi contact shift. Note that the shifts of the undistorted structure are caused by very similar spin transfer mechanisms as in the distorted structure (Figure 3). While there are no long and short bonds in the undistorted structure, there are still two Ni d_*z*^2^_–O p_*z*_ orbitals with strong overlap pointing toward each Li ion, as well as four Ni d_*z*^2^_–O p_*x*/*y*_ orbitals with weak overlap, and the same polarizing orbitals as in the distorted structure. As the material becomes less distorted, the Ni d_*x*^2^–*y*^2^_ orbital is populated at the expense of the Ni d_*z*^2^_ orbital (0.5 electrons are expected in each orbital), *i.e.*, less spin transfer is expected to occur *via* Ni d_*z*^2^_–O p_*z*_–Li s interactions and more *via* Ni d_*x*^2^–*y*^2^_–O p_*x*/*y*_–Li s interactions. The experimental spectrum is averaged according to the time the Li ions spend in each configuration. Static DFT calculations of the three structures can therefore be used to predict the NMR shifts of the Li in different defect-free structures, and the experimental spectrum will be formed from an average of the relevant subset of Li shifts, appropriately weighted also with the shifts predicted for Li near defects. This calculation implicitly assumes that motion occurs on a time scale that is faster than the NMR time scale—here, the difference in hyperfine shifts between different orientations/configurations. We leave for a future study to explore how the domain dynamics affect the NMR peak positions and line shapes as a function of temperature.

The room-temperature-predicted shifts are strongly dependent on the Curie–Weiss constants used to scale the 0 K DFT calculations, and the magnetic properties, at least at low temperatures, have been shown to be affected by the defects present in LNO. However, the observation of the main resonance at a shift position (745 ppm) that is lower than that predicted for the lowest energy zigzag structure (*P*2_1_/*c*; 760 ppm) and undistorted LNO (*R*3̅*m*; 860 ppm) suggests that collinear domains are present (*C*2/*m*; 552 ppm) at least for short time periods.

We note that we scaled the DFT calculations to 320 K, close to the estimated sample temperature for an experiment nominally performed at room temperature. Variable temperature experiments performed at close to ambient (measured temperatures) yielded a change in the shift of approximately −2 ppm/K for the main resonance (see the SI). Thus, errors in the actual sample temperature may imply slight differences in the relative contributions of the zigzag *vs.* collinear structures. The experimental and DFT errors are, however, of the same order of magnitude,^27^ and further analysis of the variable temperature NMR data will be the subject of future work.

### Antisite Mixing

The first resonance that needs to be explained in the experimental NMR spectrum of LNO is the weak resonance at –87 ppm (Figure 2a). Based on the analysis for pristine LNO, only a Li environment with exclusively 90° Li–O–Ni interactions would result in a negative shift, and this is only possible if Li is present in the Ni layer as Li_Ni_. This Li_Ni_ defect will, in turn, affect the local environment of Li in the Li layers both above and below the defect along **c** and will effectively serve to replace the bond-pathway contribution of a paramagnetic Li–O–Ni^3+^ with Li–O–Li_Ni_. Nguyen et al. have similarly ascribed the negative resonance to Li in the Ni layer using bond-pathway contributions calculated for the antisite defect-free material.^32^ To quantitatively explore the impact of point defects, we constructed four 2 × 2 × 2 supercells of the *P*2_1_/*c* zigzag LNO cell and introduced different types of defects: (i) a single Ni_Li_, (*i.e.*, Ni excess), (ii) a single Li_Ni_, (iii) Ni_Li_ and Li_Ni_ with a 180° interaction *via* O, referred to in the following as (Ni_Li_–Li_Ni_)_180_, and (iv) Ni_Li_ and Li_Ni_ separated such that they have no nearest-neighbor or next-nearest-neighbor interactions ((Ni_Li_–Li_Ni_)_sep_). In all cases, charge-neutral simulation cells were used, *i.e.*, the defect charges were compensated either directly through the complementary antisite defect (*e.g.*, Ni_Li_ has a positive relative charge compared to Li_Li_, which is compensated by the negative relative charge of Li_Ni_; with charges relative to the charge at the site in the defect-free material), or compensated electronically in the case of Ni or Li excess, where only one type of ionic defect (Li_Ni_ or Ni_Li_) is present. The size of this simulation cell was chosen so that for one defect/defect pair per simulation cell, the resulting defect concentration is close to that found in previous NMR studies of excess Ni (6%)^49^ and of the same order of magnitude as the antisite concentration in the sample studied here (approximately 3%). This cell slightly overestimates the ordering of the defects as the defects/defect pairs occupy the same position in the periodic images of the simulation cell but is used here as a starting point with which to understand and predict experimentally relevant NMR shifts. Between the defect pairs separated and in 180° configuration, the (Ni_Li_–Li_Ni_)_180_ defect pair is predicted to be energetically more favorable (Δ*E*_form,180°_ = 0.72 eV) than the defects separated from each other in the same simulation cell (Δ*E*_form,sep_ = 0.96 eV). This yields a defect association energy of Δ*E*_assoc(180-sep)_ = Δ*E*_form,180_ – Δ*E*_form,sep_ = −0.24 eV. Calculations performed with a fifth supercell containing Ni_Li_ and Li_Ni_ with a 90° interaction *via* O exhibited difficulties in converging, presumably being energetically less favorable at this defect concentration. We now use the shifts calculated using these cells to interpret our experimental NMR spectra and rationalize these based on the spin density and charges observed.

**Figure 4:**
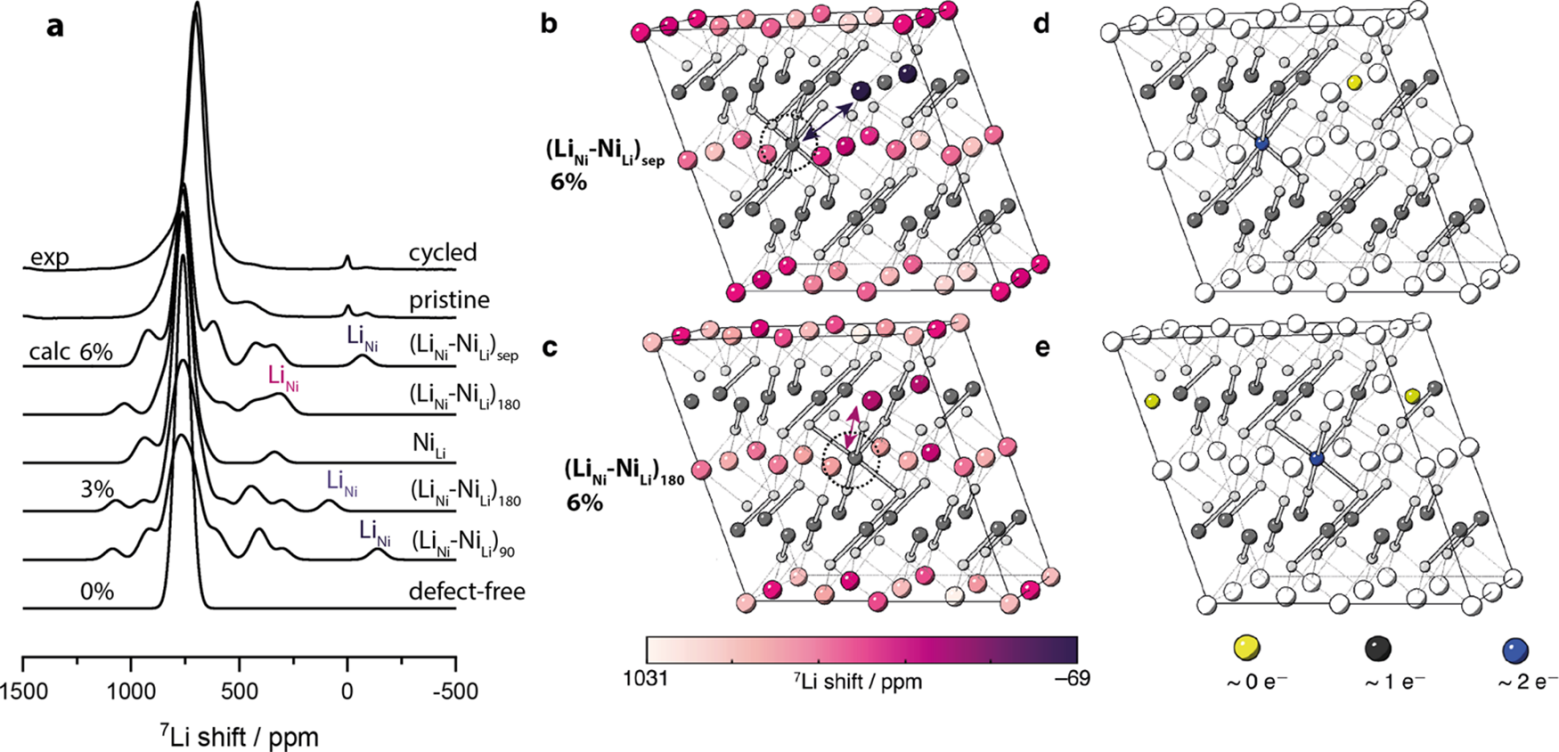
(a) Experimental ^7^Li MAS NMR MATPASS spectra of cycled and pristine LNO sample (containing 3% antisite defects) (top two spectra) and spectra calculated for various types of antisite mixing for ∼6, 3, and 0% defects (bottom six spectra). (b) Geometry-optimized zigzag supercell containing separated (*i.e.*, not nearest- or next-nearest-neighbor) antisite defects and (c) next-nearest-neighbor antisite defects, at 6% defect concentration. The Li are colored by their shift from purple (−69 ppm) to white (1031 ppm), Ni in dark gray, O in light gray, Ni–O bonds with *r*_Ni–O_ > 2 Å are shown. The position of Ni_Li_ is highlighted by the dashed circles. In parts (d, e), we reproduce the structures in parts (b, c), respectively, but the Ni ions are now colored according to the calculated magnetic moments from 0 unpaired electrons (yellow) to two unpaired electrons (blue).

The expected ^7^Li NMR spectra for cells with ∼6% of antisite defects (*i.e.*, 1 Li_Ni_ and 1 Ni_Li_ in the 2 × 2 × 2 LNO supercell) and with Ni excess (1 Ni_Li_ in the supercell) are shown in Figure 4a. The same main resonance, around 760 ppm, is predicted for all scenarios, with additional peaks emerging *ca.* 150 ppm away on either side of the main resonance. A distinct peak is seen at around −69 ppm in the case of the separated antisite defect (Ni_Li_ – Li_Ni_)_sep_, due to the lithium in the nickel layer, along with additional peaks at around 380 and 420 ppm due to nearby Li in the Li layer (Li_Li_). A peak is seen at around 360 ppm for Li_Ni_ in the case of the next-nearest-neighbor 180° interaction (Ni_Li_–Li_Ni_)_180_. Introduction of a single Ni_Li_ (as found in Ni-excess materials) generates a range of different Li_Li_ local environments with resonances at *ca.* 950 ppm and a weaker resonance at *ca.* 350 ppm, consistent with the DFT calculations of Ngyugen.^32^ The higher frequency 950 ppm peak is consistent with the experimentally observed growth of a shoulder to higher frequencies of the main peak and a decrease in the intensity of the main peak as the Ni_Li_ concentration increases.^22,32,49^ However, the observation of a peak at 350 ppm is consistent with the peak seen at approximately 460 ppm in Ni-rich samples but does not account for the experimentally observed decrease in this peak with increased Ni_Li_ concentration (see below).

Figure 4b,c shows the geometry-optimized cells from our DFT calculations for the antisite separated and 180° antisite defects (at 6% defect concentration) and allows us to identify which Li ions exhibit a particular shift; the color of the Li ions represents their calculated chemical shift values. Ni–O bonds with a distance greater than 2 Å are plotted to highlight the long Jahn–Teller axis (∼2.1 *vs*. 1.9 Å for short Ni–O Jahn–Teller distorted bonds). We note that the Jahn–Teller distortions are largely preserved with the addition of a defect, except in the immediate vicinity of the defect. The defect creates an undistorted domain, pinning the material to the undistorted state even in 0 K calculations where the ground state of the defect-free material is distorted.^18^

The calculated magnetic moment on each of the Ni ions is shown in Figure 4d,e, colored according to the moment from yellow (zero moment) to blue (corresponding to ∼2 unpaired e^–^, *i.e.*, μ_eff_ ≈ 2.7 μ_B_). As expected, there are no unpaired spins on Li and O (these ions exhibit negligible moments of approximately 0.1 e^–^). In both antisite defect pair configurations, there are two unpaired electrons on the Ni_Li_ defect, consistent with the expectation that Ni_Li_ is in a formal oxidation state of +2. Charge compensation formally occurs *via* quenching of the magnetic moment of an additional Ni in the Ni layer (*i.e.*, it is formally Ni^4+^) that is adjacent to the Li_Ni_, presumably to minimize electrostatic repulsion. The presence of this Ni^4+^, in addition to the Ni_Li_^2+^ ion in the Li layers, affects the Li hyperfine shifts of multiple nearby Li^+^ ions, not just the Li_Ni_.

The shift of Li in the Ni layer is very sensitive to its immediate environment. Li_Ni_ experiences no 180° Ni_Ni_–O–Li delocalization interactions (Figure 3a), only 90° Ni_Ni_–O–Li polarization interactions (Figure 3c), and hence exhibits negative shifts, *e.g.*, −69 ppm when the charge-compensating Ni_Li_ is not located in the first or second cation coordination shell at a 6% defect concentration (Figure 4b). Note that Li_Ni_ is involved in five Ni_Ni_–O–Li 90° interactions, as Li_Ni_ leads to the quenching of one neighboring Ni moment in the Ni layer (to 0), which in turn does not contribute to the polarization interaction. This effect is most pronounced in the scenario of an isolated Li_Ni_ (Li_1.06_Ni_0.94_O_2_) that is not compensated by Ni_Li_, resulting in a very large and negative shift of −714 ppm as the nearest oxygen ions exhibit significant magnetic moments that enhance the polarization of the Li ion (see the SI). Returning to the experimentally more relevant scenarios, if Li_Ni_ interacts with the charge-compensating Ni_Li_ defect in a 180° configuration (*i.e.*, the Ni_Li_ is in the second cation coordination shell), the Ni_Li_–O–Li_Ni_ delocalization interaction adds strong positive shift contributions to the Li shift. The contributions are particularly large as Ni_Li_ exhibits a magnetic moment of 2.7 μ_B_ (2 unpaired e^–^*vs.* 1 unpaired e^–^ in the pristine material), enhancing spin transfer to the Li ion. This results in a net positive shift of 290 ppm for the Li_Ni_ site for the 6% defect cell, since it interacts with Ni_Li_*via* two 180° interactions, one interaction in the same cell as illustrated in Figure 4c,e, and one in the adjacent cell, *via* periodic boundary conditions. The important conclusion is that not all Li_Ni_ environments give rise to resonances with negative shifts.

The peak observed experimentally at ∼460 ppm thus comprises contributions from a variety of different Li environments created by the defects: these include, first, a Li_Ni_ ion with two or more next-nearest Ni_Li_ ions, *i.e.*, (Li_Ni_–Ni_Li_)_180_ as discussed above, second, Li_Li_ ions that are adjacent to the Li_Ni_ defect, and third, Li_Li_ ions that are adjacent to (formally) Ni_Ni_^4+^ ions with quenched magnetic moments (zero unpaired electrons), the latter two environments always contributing to the 460 ppm resonance irrespective of the relative arrangement of Li_Ni_ and Ni_Li_. In separated antisite defects, the shifts for the Li_Li_ resonances near the Li_Ni_ defect are predicted to decrease from 463 to 343 ppm, whereas the shift from the Li_Li_ site near the Ni_Ni_^4+^ increases from 408 to 450 ppm. Li ions next to Ni in the Li layer are expected to experience polarizing 90° interactions with Ni_Li_, resulting in slightly negative contributions to the shift, but these are easily dominated by spin transfer interactions.

Given that the experimental sample only contained approximately 3% of antisite defects, we next explored a simulation cell that was double the size, also with a 3% defect concentration. The larger cell reduces the multiple defect interactions across periodic boundaries, which allows us to explore different (Ni_Li_–Li_Ni_) clusters interacting *via* either 180° or 90° configurations and to evaluate the subtle changes in Fermi contact shifts of defect clusters aligned along long and short Ni–O bonds. The short and long bonds refer to the Ni–O bond length in the defect-free material, the defect creating a locally undistorted environment with essentially equal Ni–O bonds around the defect, as discussed previously.^18^ The most stable defect pairs are the (Ni_Li_–Li_Ni_) defect pairs with a Ni_Li_–O–Li_Ni_ 180° interaction aligned along a short Ni–O bond direction and (Ni_Li_–Li_Ni_) pairs in a 90° configuration that are aligned along a long Ni–O bond direction in the defect-free cell (predicted spectra in Figure 4a and the energetics of the different defect clusters in the SI, Figure S7). A Li_Ni_ peak is predicted at *ca.* −130 ppm for the 90° long interaction, the corresponding 90° short configuration giving a Li_Ni_ resonance at −90 ppm (see the SI), both environments being consistent with the experimentally observed peak at negative frequencies. Furthermore, since LNO is predicted to undergo rapid dynamic changes between Jahn–Teller distorted and undistorted domains at room temperature,^18^ the short and long configurations serve as approximate shift ranges for the different defect pairs in the dynamic system at room temperature. Note that the undistorted simulation cell cannot be geometry optimised in 0 K DFT calculations as it is not the ground state: it relaxes into the distorted ground state on optimization. The 180° configuration now gives a Li_Ni_ resonance at +80 ppm for the 180° short defect arrangement and −30 ppm for the 180° long arrangement (see the SI), these configurations no longer containing more than one Li–O–Ni 180° pathway in more than one simulation cell. Thus, configurations where Li_Ni_ is compensated by Ni_Li_ but are either further away from Ni_Li_ or in a 90° Li_Ni_–Ni_Li_ configuration give rise to shifts that are consistent with the experimentally observed resonance at −87 ppm, corresponding to approximately 1% of the total Li content in this sample. Every 180° Ni_Li_–O–Li_Ni_ interaction shifts the Li_Ni_ resonance to more positive values, ultimately contributing to the 450 ppm resonance at high concentrations of (Li_Ni_–Ni_Li_) pairs, where multiple 180° Ni_Li_–O–Li_Ni_ interactions transfer spin density to a Li ion. Taking the XRD estimate of 3% antisite defect pairs and accounting for the intensity of the peak at negative frequencies (1%), 2% of all Li are expected to be Li_Ni_ in environments with multiple Ni_Li_ 180° interactions and contribute to the 460 ppm resonance.

Li_Li_ configurations nearby Ni_Li_ ions will either contribute to the experimentally observed resonance at 460 ppm or result in shoulders to high and low frequencies of the main “defect-free” LNO resonance (Figure 4a). The calculated intensities of these peaks, even in the 3% antisite model, are higher than seen experimentally (*e.g.*, 5% for the 460 ppm peak), and we ascribe this to our simple model in which the “holes” that create the Ni^4+^ ions are static; in practice, these will likely hop over multiple, similar nearby local environments. The DFT shift predictions rely on a single snapshot of the structure, including the electronic structure, while in the experiment, any dynamic electronic processes, such as electron hopping and the dynamic averaging of the Jahn–Teller distortions, will result in the lithium ion experiencing multiple local (electronic) environments. An average shift, weighted according to the time the Li ion spends in each configuration, will result as discussed above for the stoichiometric compound. To illustrate by example, if the shift of a Li_Li_ ion nearby one (formally) Ni^4+^ ion in its second coordination shell is compared to Li_Li_ in stoichiometric (all Ni^3+^) LNO, then by a simple bond-pathway analysis, this would lead to the removal of one 180° Li–O–Ni^3+^ bond pathway and a reduction of the overall hyperfine shift. This is reflected in the predicted shifts, for example, of the separated antisite defects in the 6% cell, where Li_Li_ ions near one (formally) Ni^4+^ ion exhibit shifts of 324, 352, 425, and 433 ppm (see Table S2 in the SI) *vs.* a shift of 760 ppm of a Li_Li_ ion surrounded by all (formally) Ni^3+^ ions in the defect-free simulation cell. A hopping process would likely reduce the magnitude of the shift difference between these two configurations. Note that our assumption that the relative intensities of the different LNO resonances correspond directly to the relative concentrations of different configurations assumes that the different signals are not associated with very different (short) *T*_1_/*T*_2_ times and that we are not missing a subset of Li local environments; this assumption is supported by relaxation measurements (see below).

**Figure 5:**
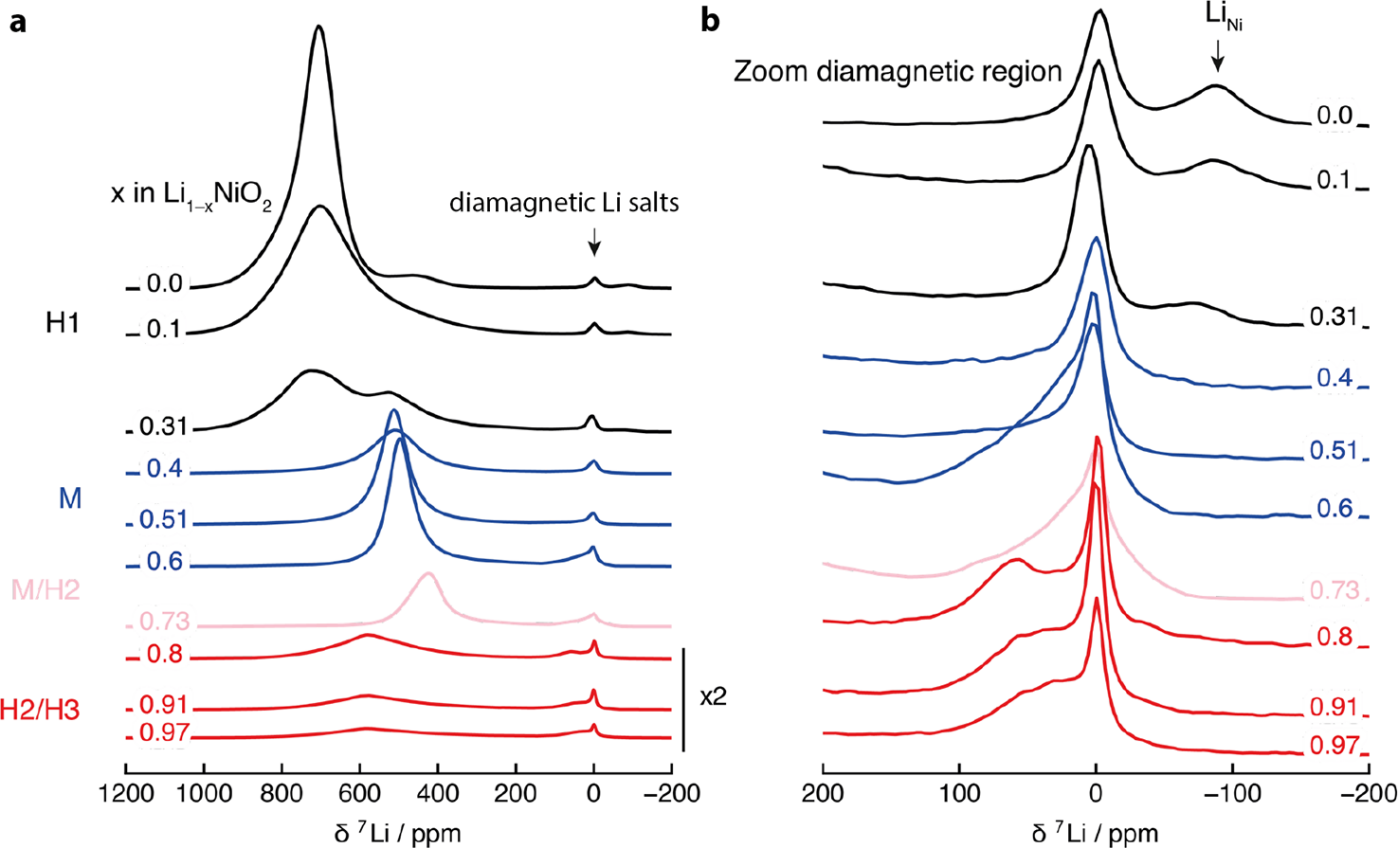
(a) *Ex situ*^7^Li MAS NMR spectra of Li_1–*x*_NiO_2_ at different states of charge, with *x* determined from the electrochemical data. The crystallographic phases determined from X-ray diffraction data are marked. The data for *x* > 0.75 are magnified 2-fold to highlight features in the spectrum. (b) The diamagnetic region is enlarged to highlight the negative peak (attributed to Li_Ni_ either distant from the charge-compensating Ni_Li_ or with these Ni_Li_ in nearest-neighbor configurations) and diamagnetic signals (δ < 200 ppm) at high states of charge. Shown here are the central slices of projection MATPASS spectra.

Li et al. have previously assigned a resonance they observed around 425 ppm to a Ni-rich rocksalt layer.^37^ Given that this is seen in as-synthesized materials where the rock salt is present in low concentrations and that we observe the 460 ppm resonance to vanish on cycling (Figure 5–see below), while rocksalt layers typically grow on cycling, this assignment appears unlikely. Nguyen et al. ascribe the 450 ppm resonance to Li environments in the vicinity of twin boundaries.^32^ We suggest that, while these twin boundaries are present to different degrees in different LNO samples and likely give rise to resonances in this frequency range, as predicted in the previous work, at least in samples that are close to stoichiometric as studied here but have antisite defects (rather than being Ni-rich), the 450 ppm peak must also contain a contribution from the antisite defects themselves.

Our predicted spectra at varying concentrations of excess Ni agree very well with the experimental ^7^Li NMR spectra reported by Karger et al., at varying levels of Ni excess,^49^ quantitively capturing the continuous decrease in intensity of the main resonance and increase in relative intensity of the higher frequency shoulder (see Figure S8a). Taking a closer look at their experimental ^7^Li NMR spectra, a small peak is seen at negative frequencies for 2.9% Ni excess that is not seen at 5.6%.^49^ In light of our shift predictions, this suggests that the 2.9% Ni samples still contain Li_Ni_ defects (with associated resonances at negative frequencies). Li_Ni_ sites with multiple Li_Ni_–O–Ni 180° interactions may also contribute to the 460 ppm resonance. The gradual decrease in the intensity of the 450 ppm peak as Ni content (and thus Li deficiency) increases further, seen in at least two studies,^32,49^ is ascribed (at least in part) to the concomitant decrease in Li_Ni_ sites.

The most important conclusion that emerges from all of the predicted spectra is that the introduction of a single defect/defect pair affects multiple nearby Li ions, causing a large number of resonances that are shifted away from the main resonance of the defect-free material at 760 ppm. The experimentally observed asymmetry of the main resonance results, at least in part, from the presence of antisite defects. Only Li_Ni_ with little or no spin transfer from nearby Ni_Li_ defects give rise to shifts that are consistent with the experimentally observed resonance at −87 ppm.

#### *Ex Situ*^7^Li NMR of Charged LNO

LNO was charged up to 4.3 V and held for 12 h to allow equilibration (see the SI for electrochemical data). The electrochemical signatures of the expected structural phase transformations (H1-M-H2/H3)^1,7,35,36^ can clearly be seen as plateaus in the voltage curve and as peaks in the d*Q*/d*V vs.* voltage plots (see the SI). Samples were then charged to specific upper cutoff voltages, and in-house X-ray diffraction measurements were performed to confirm the crystallographic structure(s) for each sample, the latter being extracted *via* Rietveld refinement at each composition (see the SI). *Ex situ*^7^Li MAS NMR measurements were then performed on the same samples (Figure 5).

Clear changes can be seen in the ^7^Li NMR spectra as a function of delithiation. These do not simply (linearly) track the change in the (formal) average oxidation state of the Ni ions neighboring Li, as also seen and discussed in previous ^7^Li NMR studies.^22,23,37^ The hyperfine shifts should, however, be informative about the structures that form, including the ionic charge and spin states, and their dynamics, and we now analyze the NMR spectra of each successive phase. In-depth theoretical calculations of the expected shifts for these phases are ongoing and are beyond the scope of this study. Note that Li et al. recently showed that the spectra obtained on delithiation were similar to those obtained on lithiation;^37^ hence, we only analyze the samples at different states of charge and after one complete cycle.

#### H1 Phase

**Figure 6:**
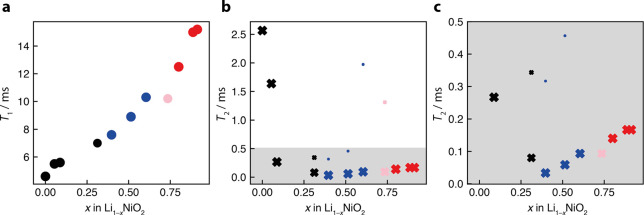
Relaxation times of Li_1–*x*_NiO_2_ as a function of *x*. (a) *T*_1_ as a function of delithiation, (b) *T*_2_ as a function of delithiation, with (c) magnification of *T*_2_ at higher states of delithiation. Here, *T*_2_ is the relaxation time constant describing the decay of transverse magnetization during a Hahn echo sequence. Black points correspond to H1 phases, blue to M, pink to a mixture of M/H2, and red to H2/H3. *T*_2_ measurements of samples with monoclinic phases required a two-component fit, with the data points scaled according to their relative contribution to the relaxation kinetics at a given stoichiometry.

In the (*R*3̅*m*) H1 phase, the main resonance first broadens on going from Li_1–*x*_NiO_2_, *x* = 0 to 0.1, and moves slightly to less positive shifts, as expected for an increase in the average oxidation state from paramagnetic Ni^3+^ to diamagnetic Ni^4+^ (or the reduction of the spin magnetic moment on Ni accompanying the oxidation of O^4^). The lack of a long-range structural (monoclinic) distortion is consistent with a random distribution of Li vacancies. The NMR spectrum at *x* = 0.31 exhibits a signal at around 500 ppm that, at first sight, resembles the main resonance of the monoclinic phase. No monoclinic (second) phase is seen, however, by XRD (see the SI). The peak position of this new resonance is in line with what might be expected for a sample where approximately 30% of the Ni^3+^ is oxidized to Ni^4+^. What is perhaps more surprising is that a resonance close to the peak position of the original LNO (*x* = 0) signal remains. Furthermore, the overall decrease in ^7^Li intensity is more pronounced than expected based on the Li extracted. We ascribe this to a concomitant drop in the *T*_2_ relaxation times, presumably due to an increase in Li mobility, the mobility being on the order of the frequency separation between the environments the Li ion is hopping between.^52,53^ This is supported by *T*_2_ MAS NMR measurements (Figure 6), the *T*_2_ dropping from 2.6 ms for LNO to 0.08 ms for Li_0.69_NiO_2_. Note that the *T*_2_ measurements of samples containing monoclinic phases required a two-component fit, indicative of two sites with distinct relaxation kinetics in the monoclinic structure. The size of the data points in the *T*_2_ plot is scaled according to their respective contribution to the relaxation at a given stoichiometry. Qualitatively similar trends are observed in both NMC–811 and NCA.^25,26,54^

Of note, the spin–lattice relaxation, *T*_1_, times gradually lengthen (with one exception at *x* = 0.73), tracking the decrease in paramagnetic ion concentration. While a detailed analysis of the *T*_1_/*T*_2_ relaxation times of all of the different pristine LNO signals was not attempted, since Hahn echoes, rather than MATPASS, experiments were performed, similar or longer relaxation times were obtained for the minority peaks.

The onset of fast Li-ion dynamics for *x* > 0.25 can be attributed to the formation of divacancies. For vacancy-ordered structures of Li_0.75_NiO_2_ (with both *P*2/*m* and C2/*m* symmetry)—first described by Arroyo y de Dompablo et al.—the vacancies were found *via* DFT calculations to exist as monovacancies to minimize repulsion.^43^ Beyond this degree of delithiation, divacancies begin to form by default, thus enabling fast Li migration.^55^ Assuming a random solid solution, the probability that a Li^+^ ion is adjacent to a double vacancy is 6*x*^2^; only half these divacancy configurations are involved in the octahedral-tetrahedral-octahedral jump processes, but at *x* = 0.31, the probability is now high (0.29), and extremely rapid motion might be expected. The NMR spectra of the delithiated phase seen experimentally are consistent with Li environments rich in Ni^3+^ similar to those found in LNO, and not more Ni^4+^ rich environments, suggesting some vacancy ordering, at least locally.

With increasing delithiation of the H1 phase, the resonances assigned to Li_Ni_ (and Ni_Li_) antisites in pristine LNO decrease in intensity, shift, and broaden. The shift of the residual intensity of the Li_Ni_ site (without Ni_Li_ in the second cation coordination shell) from −87 to −75 ppm (*x* = 0.31) is consistent with the oxidation of Ni_Ni_ ions in its first coordination shell. The decrease in intensity of this resonance, which is more pronounced for *x* = 0.31, could, in principle, occur because either these Li^+^ ions are removed or the Ni_Li_ ions associated with them migrate, resulting in different Li shifts. Specifically, if defect association occurs and distant Ni^2+^ ions in the lithium layer moved to form the more energetically favorable antisite defects, *i.e.*, with 180° arrangements to Li_Ni_, (Ni_Li_–Li_Ni_)_180_, this would result in the loss of the resonance at negative frequencies, Li_Ni_ ultimately contributing to the resonance at 500 ppm in Ni-rich environments. It is tempting to assign the lack of long-range vacancy ordering to the presence of defects in this structure by analogy with the lack of a long-range JT distortion in the pristine material.^18^

#### Monoclinic LNO

The *ex situ*^7^Li NMR data for the samples with 0.4 < *x* < 0.6 is shown in blue in Figure 5. All XRD patterns could be refined in the monoclinic M phase with symmetry *C*2/*m*, a phase consisting of a single Li environment with partial occupancy without constraining the Li occupancies to any Li ordering. Throughout the monoclinic phase region, even though the phase exhibits a broad range of Li contents and thus a change in average Ni formal oxidation state from +3.4 to +3.6, the change in hyperfine shift is small, varying from only 520 ppm (Li_0.6_NiO_2_) to approximately 500 ppm (Li_0.4_NiO_2_). The main resonance is narrower than in the H1 phase and has a larger Lorentzian contribution to the peak shape, suggestive of high Li mobility. A *T*_2_ minimum is seen here for Li_0.6_NiO_2_ at 0.025 ms, the *T*_2_ time then increasing to 0.1 ms for *x* = 0.6, accounting for the increase in intensity seen in this regime (despite Li loss) and indicating a change in Li motion and electron hopping on the NMR time scale.^23^

On the basis of the observed pristine LNO shift of 745 ppm and a composition of Li_0.6_NiO_2_ with approximately 40% Ni^4+^, we estimate a shift of 447 ppm. Larger shifts might be expected if we use the monoclinic zigzag structure to estimate the shift or account for the changes in cell parameters on charging. The observed shift of 520 ppm is therefore consistent with a model of Li mobility in this phase and no long-range ordering of Li ions. The signal assigned to Li_Ni_ without Ni_Li_ in the second cation coordination shell in the pristine material has either disappeared entirely or moved to more positive shifts at this SOC. A weak, broad peak emerges in Li_0.4_NiO_2_ as a shoulder (from 0 to approximately 100 ppm) to the signal from the diamagnetic Li species. It is tempting to assign it to Li in tetrahedral sites (above the vacancy formed in the Ni layers if Li_Ni_ is extracted), but it could also arise from Li nearby largely Ni^4+^ ions. It has also been assigned to Li in rocksalt phases by others.^37^ A central question requiring further experimental and computational investigation in the future is the question as to what causes the monoclinic phase and what role loss or change in the nature of the antisite defects play in the formation/suppression of the monoclinic phase.

#### H2/H3

Previous studies have reported that the H2 phases are formed on delithiation of the M phase beyond *ca.* 65–70%.^5,7^ Rietveld refinements of our sample at 73% delithiation, however, reveal coexistent M and H2 phases (see the SI). The material was subsequently charged to voltages beyond the H2/H3 transition of 4.2, 4.3, and 4.45 V with a notional Li content of *x* = 0.8, 0.91, 0.97, respectively, as determined from electrochemical capacity data. Although H3 phases only are expected on the basis of the electrochemical data, XRD always gives a mixture of H2 and H3 phases (see the SI), which we attribute to self-discharge, *i.e.*, the spontaneous loss of charge at high states of charge as a result of chemical redox processes,^56^ coupled with a difficulty of driving the phase completely to H3. Residual antisite defects and Ni_Li_ may also affect the ability of the material to undergo the large collapse of the *c*-parameter that occurs as part of the H2 to H3 transition. The ^7^Li NMR spectra of the H2/H3 samples are shown in red in Figure 5. The resonance at 600 ppm can be attributed to Li environments in the H2 phase due to its decreasing intensity with increasing SOC; this is consistent with previous work, where the hyperfine shift was rationalized in terms of the ordering of Ni^3+^ in Li_0.25_NiO_2_ to form 180° Li–O–Ni^3+^ superexchange interactions in favorable linear interlayer Li–O–Ni–O–Li configurations.^23,37,43^ Furthermore, this signal drops from *x* = 0.8 to 0.91 and 0.97, as less H2 remains and the sample is almost fully delithiated. The shoulder at 0–100 ppm sharpens, and a distinct resonance is seen at approximately 70 ppm at *x* = 0.8. Its intensity drops and a broad peak remains with a smaller hyperfine shift as *x* increases. These relatively featureless signals are reminiscent of the Li signals in the rocksalt material Li_2_NiO_2_F, which have been assigned to Li close to either diamagnetic Ni^4+^ or Li-rich environments.^57^ Again, it is difficult to definitely assign these signals to a rocksalt phase, tetrahedral Li sites, or defect structures in H2 (including grain boundaries^32^). There seems little correlation, however, between this signal and H3 content, and hence, it is unlikely to be due to Li in an ordered H3 phase.

#### Discharged LNO

Finally, the sample of LNO charged to 4.3 V, held for 12 h to allow equilibration, discharged to 3 V, and then held again for 12 h to allow for further equilibration and reinsertion of Li (see the SI for electrochemical data) was studied by NMR (Figure 4a, first two spectra, and Figure S4d). About 95% relithiation was achieved. The intensities of the resonances at 460 and −87 ppm (and the shoulder to high frequency) are reduced in comparison to pristine LNO. One possible explanation accounting for a decrease of the intensities of these resonances would be that Li_Ni_ is extracted from the Ni layer on cycling. Such a removal would be consistent with the low calculated energy barrier for interlayer (transition-metal layer to Li layer) migration of Li in Li-rich Li_2_MnO_3_ cathode materials.^58^ Rietveld refinement of the discharged sample also supports this hypothesis with Ni_Li_ = 2.3% and Li_Ni_ = 0% *vs.* Ni_Li_ = 3.8(2)% and Li_Ni_ = 3.0(3)% obtained after and before cycling, respectively. This observation could furthermore provide a partial explanation for the large first cycle capacity loss that is often reported for LNO.^6^ Removal of Li_Ni_ could also be associated with migration of the Ni_Li_ ions, again resulting in changes to the spectrum.

Our data is fully consistent with the interpretation in terms of the antisite (point) defects and does not require invoking planar defects such as twin boundaries in the analysis. As point defects typically interact with planar defects, however, *e.g.*, enrich at grain boundaries, it is highly likely that the NMR signatures of the antisite defects are affected by grain boundaries. We leave for a future study to explore the correlations between the NMR signatures of the antisite defects and the grain boundary concentrations in the material.

## Conclusions

We have used a combination of *ex situ*^7^Li NMR measurements and density functional theory calculations to revisit the local structure of LNO, including Jahn–Teller distortions, antisite mixing, and dynamics on delithiation. Our density functional theory calculations show that an average of the Jahn–Teller distorted (zigzag/collinear) and undistorted material is the best fit for our experimental ^7^Li NMR spectrum. Additional peaks present in the NMR spectrum can be attributed to antisite mixing, the resonance at negative frequencies accounting for approximately one third of the total Ni_Li_–Li_Ni_ antisite defects as determined by XRD. Specifically, this resonance corresponds to Li sites in the nickel layers (Li_Ni_) either distant from the charge-compensating Ni ions in the lithium layers or with these Ni ions in the nearest-neighbor coordination shell, these configurations being generally higher in energy than environments with Ni ions in next-nearest-neighbor positions in the lithium layer. These Li_Ni_ are either removed on cycling or rearrange into next-nearest-neighbor configurations with Ni_Li_.

On electrochemical delithiation of LNO, all X-ray diffractive and electrochemical signatures of the H1–M–H2–H3 phase transitions are seen. The *ex situ*^7^Li NMR Fermi contact shifts of the partially delithiated phases do not tend linearly to zero on delithiation, as might be expected for a system with no charge and cation ordering. . Instead, we find new environments emerging in the delithiated H1 phase, along with an abrupt decrease in signal intensity due to increased Li-ion mobility consistent with the drop in the *T*_2_ relaxation time. The observation of a single resonance with a short *T*_2_ time for the monoclinic phase is similarly consistent with fast Li-ion mobility. At high states of charge, we find a mixture of H2 and H3 phases, with the highly shifted peaks stemming from the H2 phase. The absence of an NMR signal for the H3 phase suggests this phase is devoid of Li (or Li is located in diamagnetic environments).

## Supporting Information Available

Electrochemical profiles of LNO/Li half-cells; XRD diffractograms and refinements of the delithiated phases as a function of the state of charge; phase fractions as determined with XRD; peak positions and ratios of ^7^Li MAS NMR spectra of pristine and cycled LNO at room temperature; the impact of changes in temperature on the experimental ^7^Li NMR shifts; predicted spectra of all antisite defect configurations at varying defect concentrations; impact of the choice of hybrid functional on the predicted Fermi contact shifts; and the impact of selected *k*-point mesh on the predicted Fermi contact shifts (PDF)
