# Supplementary material for: Probing Jahn–Teller Distortions and Antisite Defects in LiNiO2 with 7Li NMR Spectroscopy and Density Functional Theory
Source: Chem Mater. 2024 Apr 24;36(9):4226–39. doi: 10.1021/acs.chemmater.3c03103 (PMC11099921; doi:10.1021/acs.chemmater.3c03103)
Supplement: Supplementary file 1 — cm3c03103_si_001.pdf [file cm3c03103_si_001.pdf]

Probing Jahn–Teller Distortions and Antisite Defects in $LiNiO_2$ with ^7Li NMR Spectroscopy and Density Functional Theory

Supporting Information

*Annalena R. Genreith-Schriever[1,6§], Chloe S. Coates[1,6§], Katharina Märker[1,2,6], Ieuan D. Seymour[3,4,5,6], Euan N. Bassey[1#], Clare P. Grey[1,6]**

[1]Yusuf Hamied Department of Chemistry, University of Cambridge, Cambridge CB2 1EW, U.K.

[2]Univ. Grenoble Alpes, CEA, IRIG, MEM, Grenoble 38000, France

[3]Department of Materials, Imperial College London, London SW7 2AZ, U.K.

[4]Department of Chemistry, School of Natural and Computing Sciences, University of Aberdeen, Aberdeen AB24 3FX, U.K.

[5]Advanced Centre for Energy and Sustainability, School of Natural and Computing Sciences, University of Aberdeen, Aberdeen AB24 3FX, U.K.

[6]The Faraday Institution, Harwell Science and Innovation Campus, Didcot OX11 0RA, U.K.

[*]Email: cpg27@cam.ac.uk

Electrochemistry

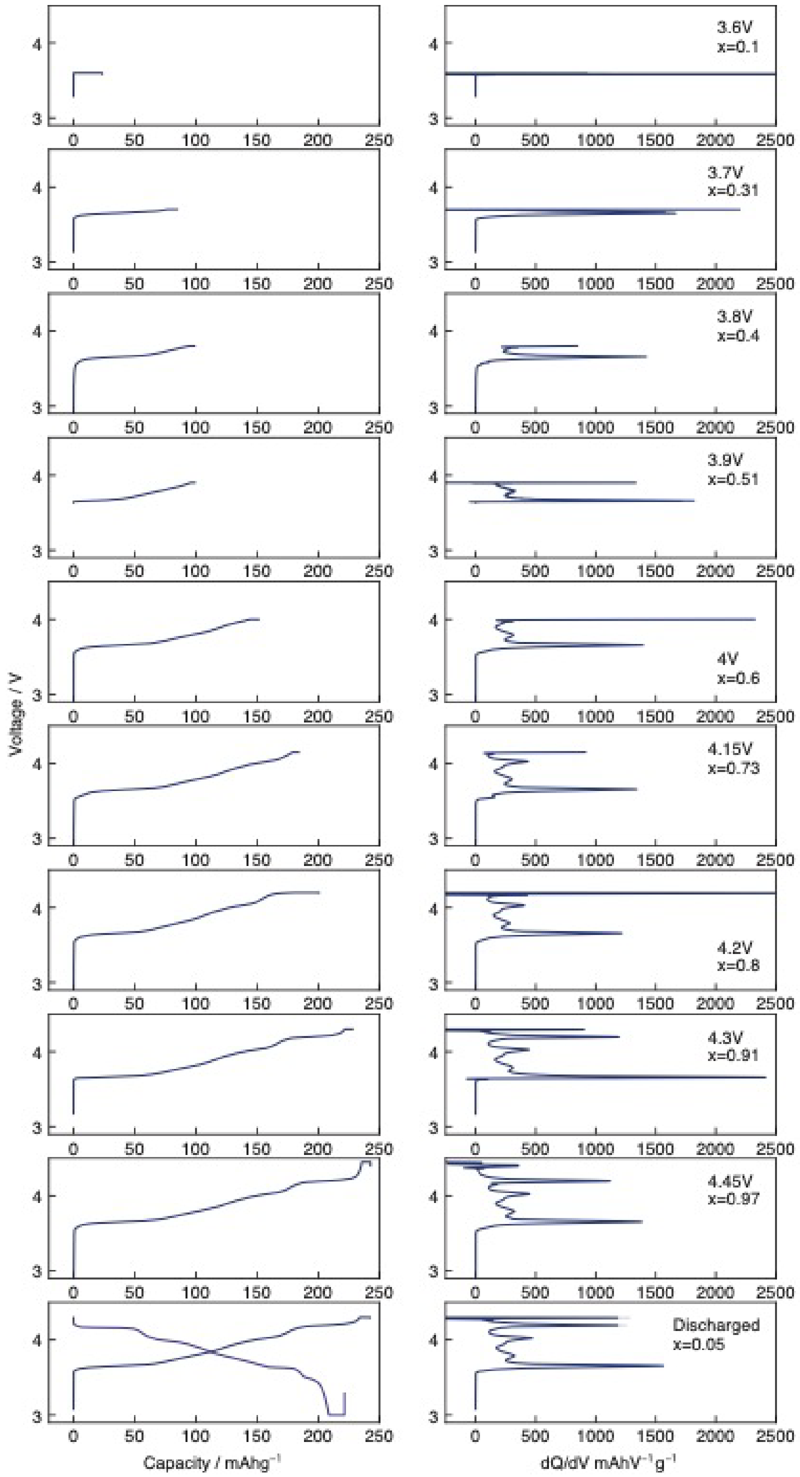

Figure S1 Electrochemical profiles for LNO:Li half cells cycled to different upper cut-off voltages at a rate of 0.1C and held for 12h.

XRD refinements

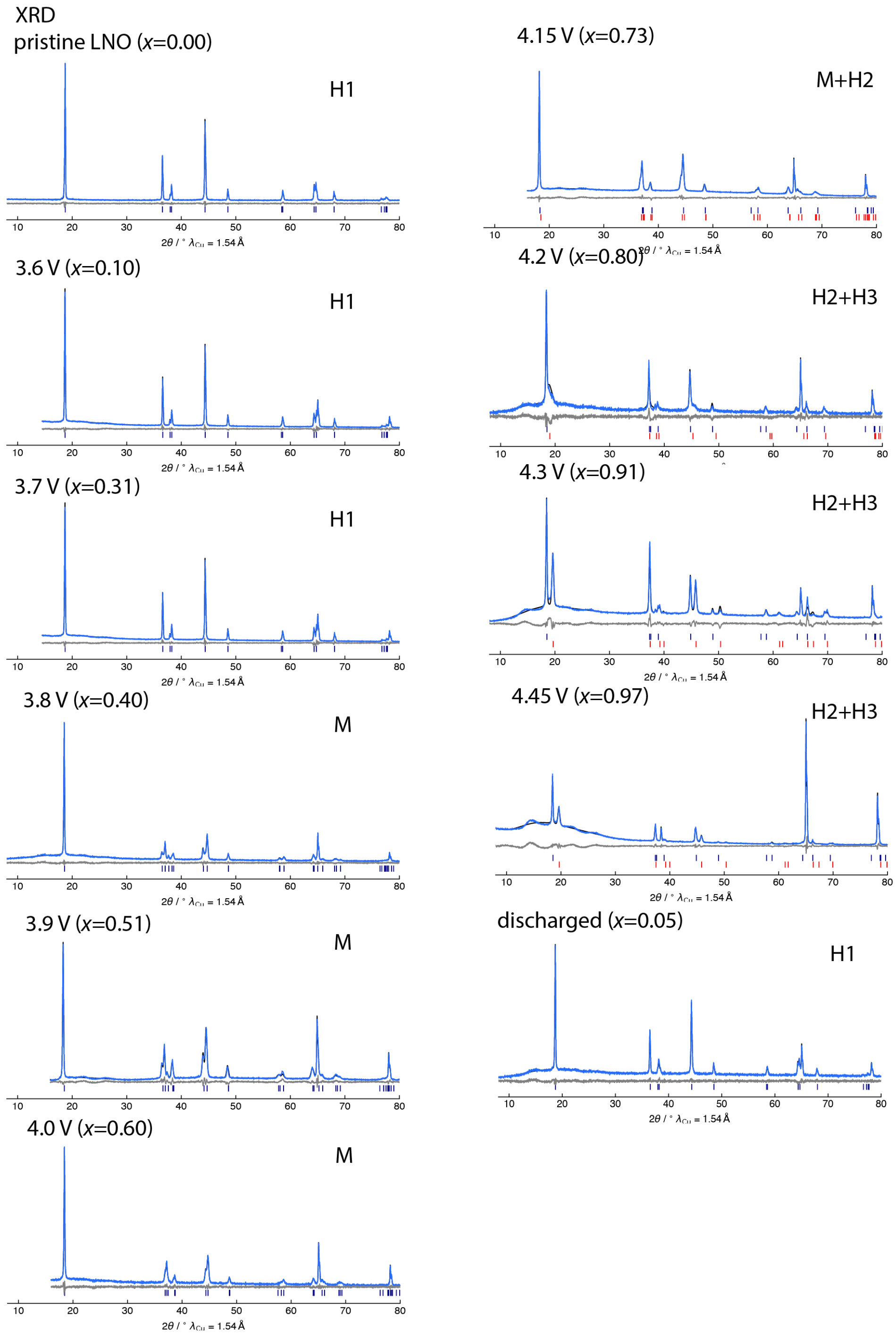

Figure S2 XRD diffractograms and refinements of the delithiated phases as a function of state of charge.

The structures discussed by Li *et al.*[1] were used as the starting point for our refinements of all four phases. It is the most recent comprehensive structural evaluation, based on earlier findings reported by Dyer *et al.*[2], Peres *et al.*[3], and Arai *et al.*[4, 5]

ICSD codes used were as follows:

H1 - R-3m - 26608

M - C2/m - 50412

H2 and H3 are the same symmetry as H1, so the same structure was used as a starting point, with lower refined Li occupancy and O positions and using the lattice parameters reported by Li *et al.*[1]

Delithiated phases as identified with XRD

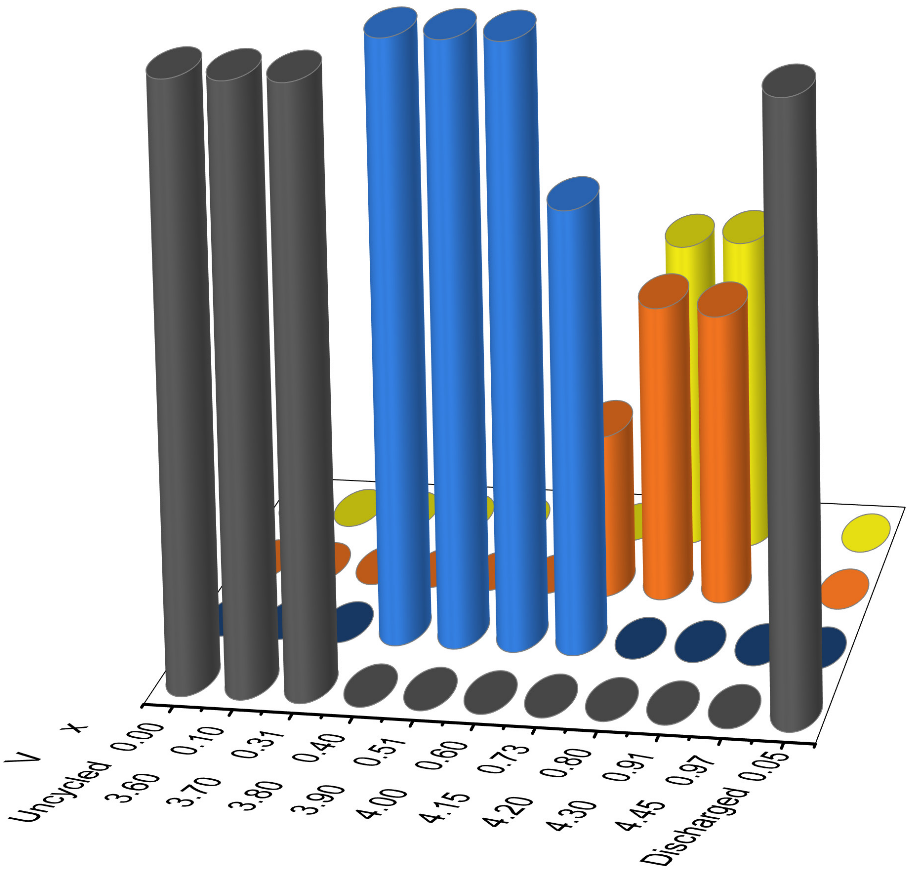

Figure S3: Phases forming on delithiation of $Li_{1-x}NiO_2$ as a function of state of charge, phase fractions determined with XRD.

Solid-state NMR

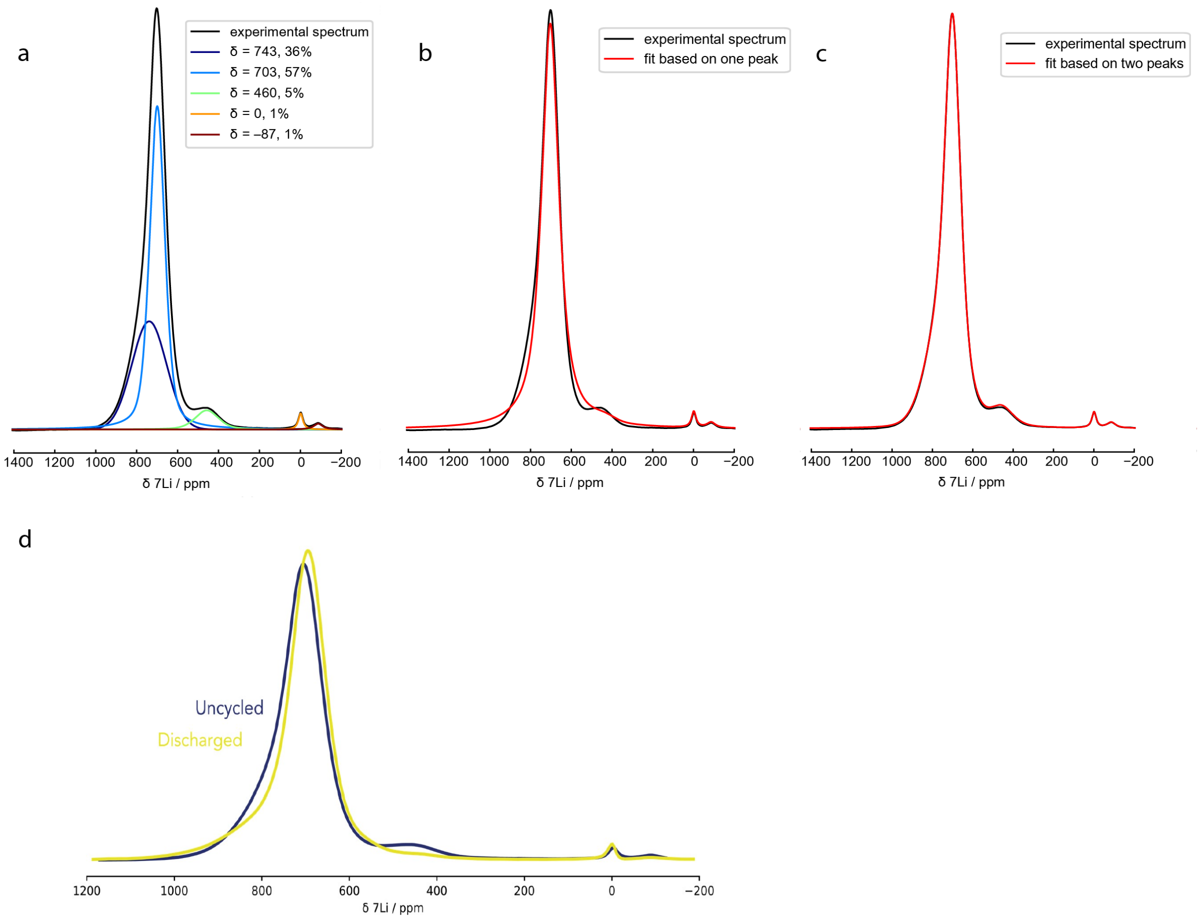

Figure S4 Experimental ^{7}Li MAS spectra of LNO at room temperature. (a) Peak positions and ratios of the pristine material; fit of the main resonance with (b) a single peak and (c) two peaks, accounting for the asymmetry of this resonance which cannot stem from one single Li environment. (d) Comparison of the spectra before and after cycling.

Table S1: Fitting parameters for ^{7}Li NMR pj-matpass spectra as fit using DMfit

Sample	Position	Intensity	G/L	Width	%
Pristine LNO	743.1	488338	1.15	199.67	36.0
Pristine LNO	703.4	1460071	0.58	88.02	57.0
Pristine LNO	460.0	86811	0.56	134.5	5.2
Pristine LNO	-1.6	73483	-0.12	134.5	1.1
Pristine LNO	-87.4	26834	-0.13	51.36	0.78
3.7V (x=0.31)	720.2	306653	1	154	21.33
3.7V	517.1	325148	-0.3	207	50.87
3.7V	5.8	143348	0	25.6	2.46
3.7V	–74.6	11027	4.86	35.2	0.18
3.7V	729.37	190969	0.92	291.77	25.2
3.7V	720.2	306653	1	154	21.3

Impact of changes in temperature on the experimental ^{7}Li NMR shifts

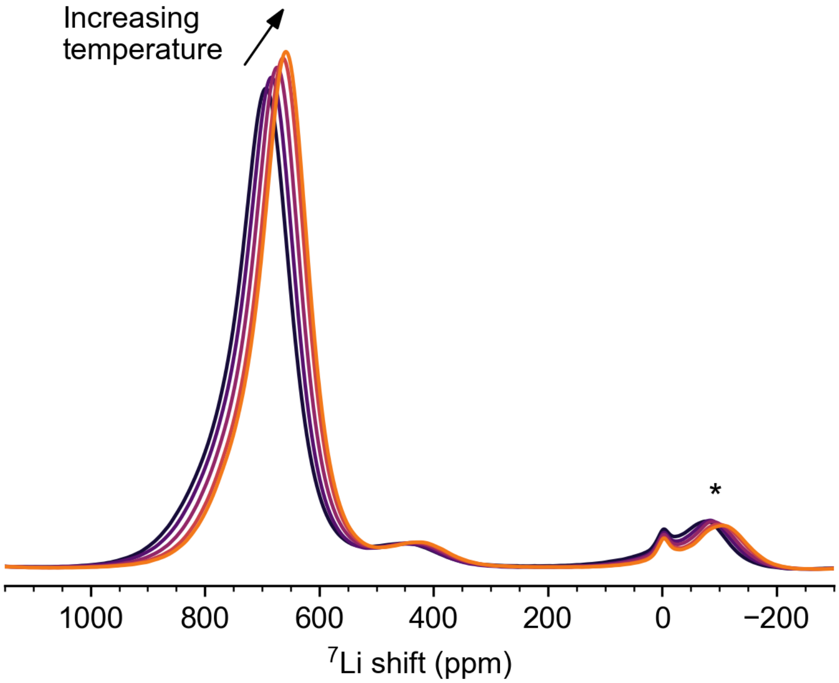

Figure S5: ^{7}Li Hahn echo spectra of $LiNiO_2$ recorded as a function of temperature at 4.7 T magnetic field strength and 60 kHz MAS frequency. The variable temperature gas flow in the 1.3 mm MAS NMR probe was heated to record spectra at sensor temperatures ranging from 25 °C (blue) to 60 °C (orange), with the ^{7}Li shift of the main signal decreasing from 693 ppm to 658 ppm within this temperature range. Note that the temperature sensor is located close to the rotor, but the actual sample temperature is considerably higher at 60 kHz MAS frequency due to significant frictional heating, typically between 50 and 55 °C at 25 °C sensor temperature and between 65 and 70 °C at 60 °C sensor temperature.

DFT calculations

6% Defects

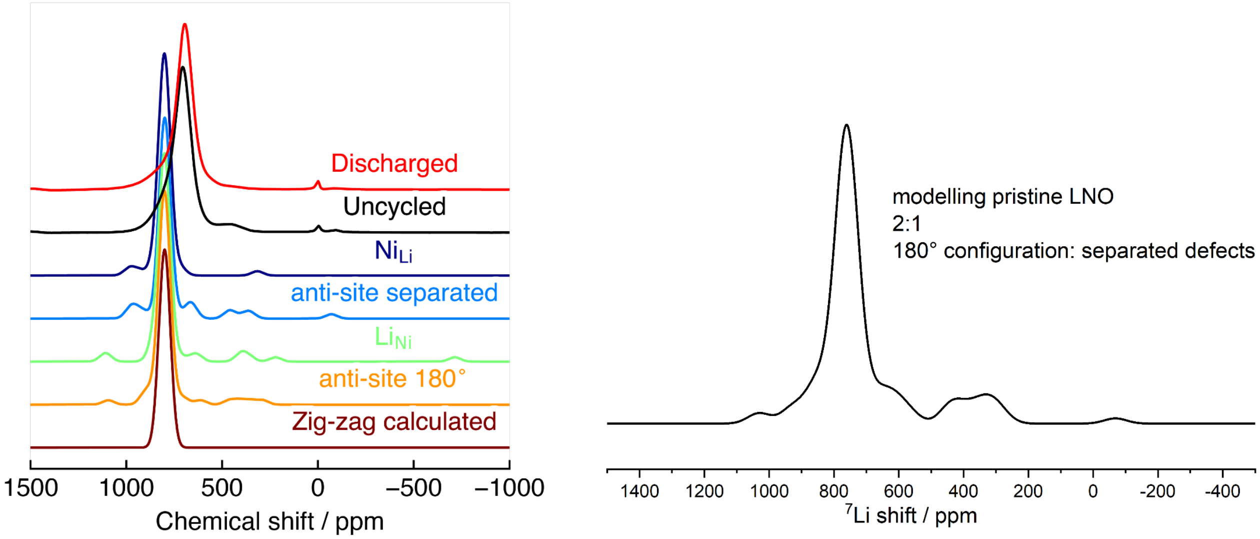

Figure S6: Comparison of experimental and predicted ^{7}Li NMR spectra for 6% defects, relating to Figure 4a of the main body, including the predicted spectra of isolated Ni_{Li} and Li_{Ni} defects. To account for the higher defect concentration in the simulations (6% *vs.* 3% in experiment), the spectra were summed according to a 1:1 ratio of the respective defect cell *vs.* a defect-free zigzag distorted simulation cell. Based on the experimental observations of 1/3 of the antisite defect pairs being separated, 2/3 assuming next-nearest neighbour configurations, and the total defect concentration amounting to 3%, a spectrum was modelled by summing over 50% defect-free zigzag distorted bulk, 33% antisite defects in a 180° configuration and 17% separated defects.

Table S2: ^{7}Li Fermi contact shifts predicted for an antisite defect pair separated from each other and in a 180° configuration at 6% defect concentration.

Predicted ^{7}Li shift / ppm	
$(Li_{Ni}-Ni_{Li})_{sep}$	$(Li_{Ni}-Ni_{Li})_{180°}$
-69	289
324	324
352	378
425	442
433	569
607	651
612	675
619	737
630	752
690	770
707	774
834	801
866	843
924	848
928	864
932	1031

3% Defects

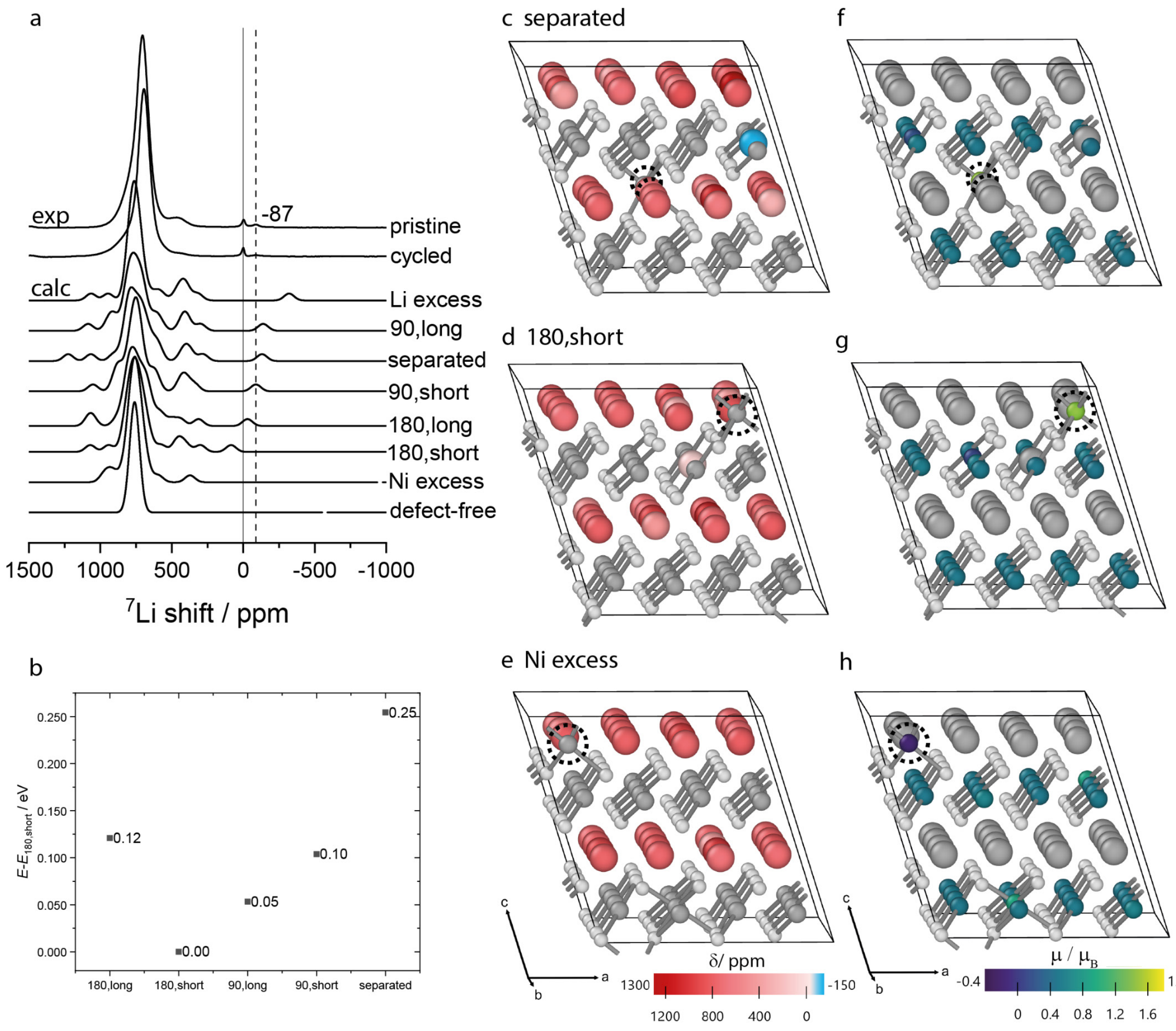

Figure S7: (a) Experimental ^{7}Li MAS NMR MATPASS spectra of cycled and pristine (3% anti-site defects) LNO (top two spectra) and spectra calculated for various types of antisite mixing for ~3% defects and the defect-free material (bottom eight spectra). (b) Energy of the defect pairs relative to the most stable 180,short configuration. (c) The geometry-optimised zigzag supercell containing separated (*i.e.* not nearest- or next-nearest neigbour) antisite defects, (d) next-nearest neighbour antisite defects, and (e) Ni excess. The Li are coloured by their shift from blue (-150 ppm) to red (1300 ppm), Ni in dark grey, O in light grey, Ni–O bonds with $r_{Ni-O} > 2$ Å are

shown. The position of Ni_{Li} is highlighted by the dashed circles. In (f)-(h) we reproduce the structures in (c)-(e), respectively, but the Ni ions are now coloured according to their spin magnetic moments corresponding to 0 unpaired electrons (purple) to 2 unpaired electrons (yellow). Note that the moment of Ni_{Li} is decreased drastically when there is no compensating Li_{Ni} defect in the same simulation cell (h), corresponding to less than one unpaired electron as compared to the two unpaired electrons seen on Ni_{Li} when Li_{Ni} is in the same cell (f,g).

Impact of varying defect concentration on the predicted spectra

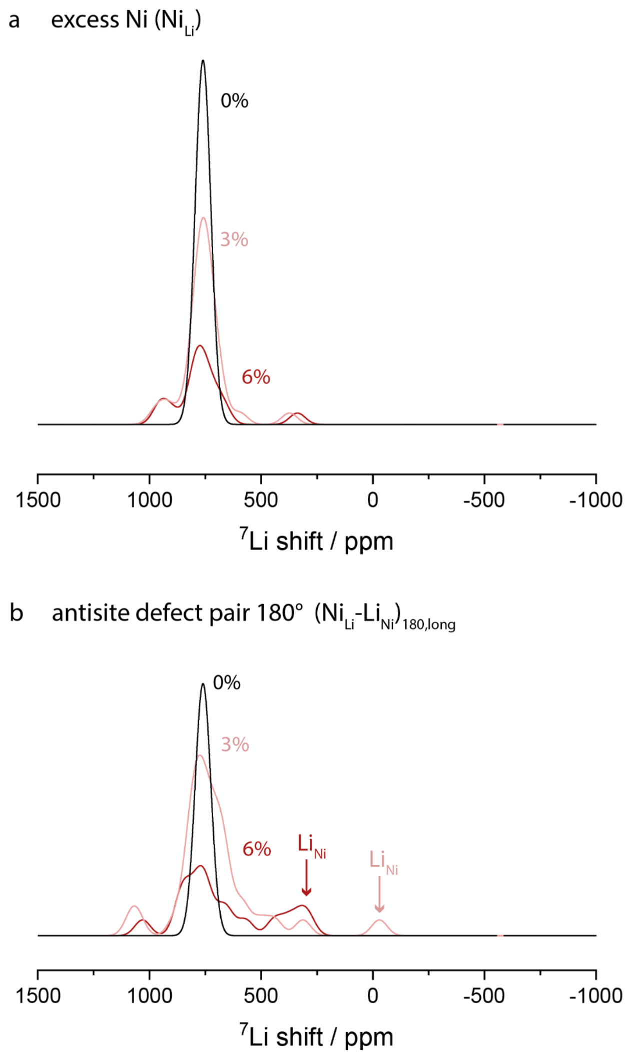

Figure S8: Impact of defect concentrations on the ^{7}Li NMR spectra of (a) excess Ni and (b) an antisite defect pair in next-nearest neighbour 180° configuration (the 3% defects aligned along a long Ni-O bond in the defect-free structure, the 6% defects aligned along a long Ni-O bond but interacting along short Ni-O bonds across periodic boundary conditions), as predicted with 2x2x2 and 2x4x2 simulation cells for 6% and 3% antisite defects, respectively. The Ni excess spectra in (a) agree very well with the experimental spectra reported by Karger *et al.*[6] The shift of the Li$_{Ni}$ 180° defect in (b) is very sensitive to the defect concentrations, shifting from -30 ppm at 3% to +290 ppm at 6%.

Functionals analysis

Kim *et al.* have previously reported a Fock exchange of 20% to yield the most reliable hyperfine shifts.[7] Varying the hybrid functional from B3LYP to HSE06 and PBE0 all at 20% exchange had negligible impact on the shifts (see Figure S9), whereas a variation of the Fock exchange from 20% to 35% caused > 100 ppm differences in the shifts of zigzag distorted $LiNiO_2$.

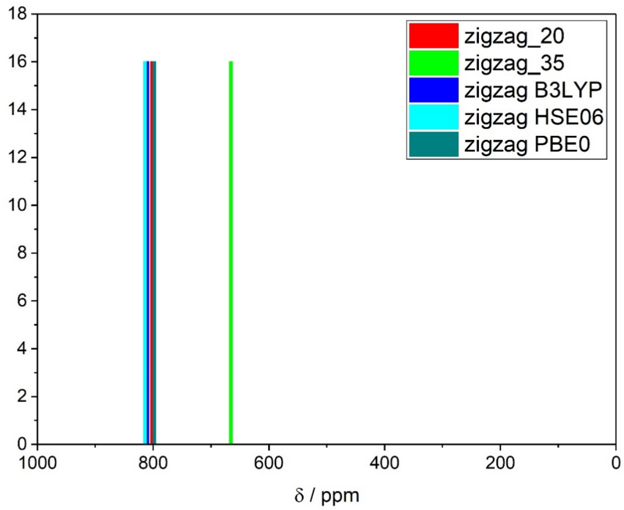

Figure S9: Comparison of different hybrid functionals (B3LYP, HSE06, and PBE0, all at 20% Fock exchange) and amounts of Fock exchange (20% *vs.* 35%). The impact of the choice of functional is negligible.

***k*-point analysis**

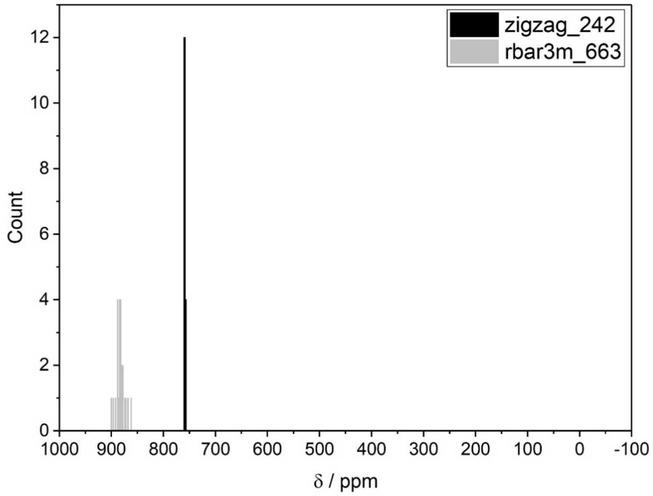

Figure S10: Predicted ^{7}Li Fermi contact shifts at varying *k*-point meshes. $R\bar{3}m$ shifts are predicted to be *ca.* 2,000 ppm when using the default *k*-point mesh (2x2x2).[8] When increasing the *k*-point density (*e.g.*, to 6x6x3), the predicted shifts decrease and become more comparable to the shifts of the distorted structure (870 ppm $R\bar{3}m$ *vs.* 760 ppm for the distorted structure).

References

(1) Li, H.; Zhang, N.; Li, J.; Dahn, J. R. Updating the Structure and Electrochemistry of Li_xNiO_2 for $0 \leq x \leq 1$. *J. Electrochem. Soc.* **2018**, *165* (13), A2985. DOI: 10.1149/2.0381813jes.

(2) Dyer, L. D.; Borie Jr, B. S.; Smith, G. P. Alkali metal-nickel oxides of the type $MNiO_2$. *J. Am. Chem. Soc.* **1954**, *76* (6), 1499-1503. DOI: 10.1021/ja01635a012.

(3) Peres, J. P.; Demourgues, A.; Delmas, C. Structural investigations on $Li_{0.65-z}Ni_{1+z}O_2$ cathode material: XRD and EXAFS studies. *Solid State Ionics* **1998**, *111* (1), 135-144. DOI: 10.1016/S0167-2738(98)00122-2.

(4) Arai, H.; Okada, S.; Ohtsuka, H.; Ichimura, M.; Yamaki, J. Characterization and cathode performance of $Li_{1-x}Ni_{1+x}O_2$ prepared with the excess lithium method. *Solid State Ionics* **1995**, *80* (3), 261-269. DOI: 10.1016/0167-2738(95)00144-U.

(5) Arai, H.; Tsuda, M.; Saito, K.; Hayashi, M.; Takei, K.; Sakurai, Y. Structural and Thermal Characteristics of Nickel Dioxide Derived from $LiNiO_2$. *J. Solid State Chem.* **2002**, *163* (1), 340-349. DOI: 10.1006/jssc.2001.9428.

(6) Karger, L.; Weber, D.; Goonetilleke, D.; Mazilkin, A.; Li, H.; Zhang, R.; Ma, Y.; Indris, S.; Kondrakov, A.; Janek, J.; Brezesinski, T. Low-Temperature Ion Exchange Synthesis of Layered $LiNiO_2$ Single Crystals with High Ordering. *Chem. Mater.* **2023**, *35* (2), 648-657. DOI: 10.1021/acs.chemmater.2c03203.

(7) Kim, J.; Middlemiss, D. S.; Chernova, N. A.; Zhu, B. Y. X.; Masquelier, C.; Grey, C. P. Linking Local Environments and Hyperfine Shifts: A Combined Experimental and Theoretical ^{31}P and ^{7}Li Solid-State NMR Study of Paramagnetic Fe(III) Phosphates. *J. Am. Chem. Soc.* **2010**, *132* (47), 16825-16840. DOI: 10.1021/ja102678r.

(8) Middlemiss, D. S.; Ilott, A. J.; Clément, R. J.; Strobridge, F. C.; Grey, C. P. Density Functional Theory-Based Bond Pathway Decompositions of Hyperfine Shifts: Equipping Solid-State NMR to Characterize Atomic Environments in Paramagnetic Materials. *Chem. Mater.* **2013**, *25* (9), 1723-1734. DOI: 10.1021/cm400201t.
